# Supplementary material for: Selected reaction monitoring for the quantification of Escherichia coli ribosomal proteins
Source: PLoS One. 2020 Dec 14;15(12):e0236850. doi: 10.1371/journal.pone.0236850 (PMC7735604; doi:10.1371/journal.pone.0236850)
Supplement: S1 Fig — Candidate peptides for selected reaction monitoring analysis were selected using Skyline software. We quantified the prepared r-proteins using purified ribosomes, E. coli lysate, and r-protein-overexpressed E. coli lysates using a liquid chromatograph-triple quadrupole mass spectrometer. (PDF) [file pone.0236850.s001.pdf]

**S1 Fig. Ion chromatograms corresponding to peptides derived from r-proteins.**
Candidate peptides for selected reaction monitoring analysis were selected using Skyline software.
We quantified the prepared r-proteins using purified ribosomes, *E. coli* lysate, and r-protein-overexpressed *E. coli* lysates using a liquid chromatograph-triple quadrupole mass spectrometer.

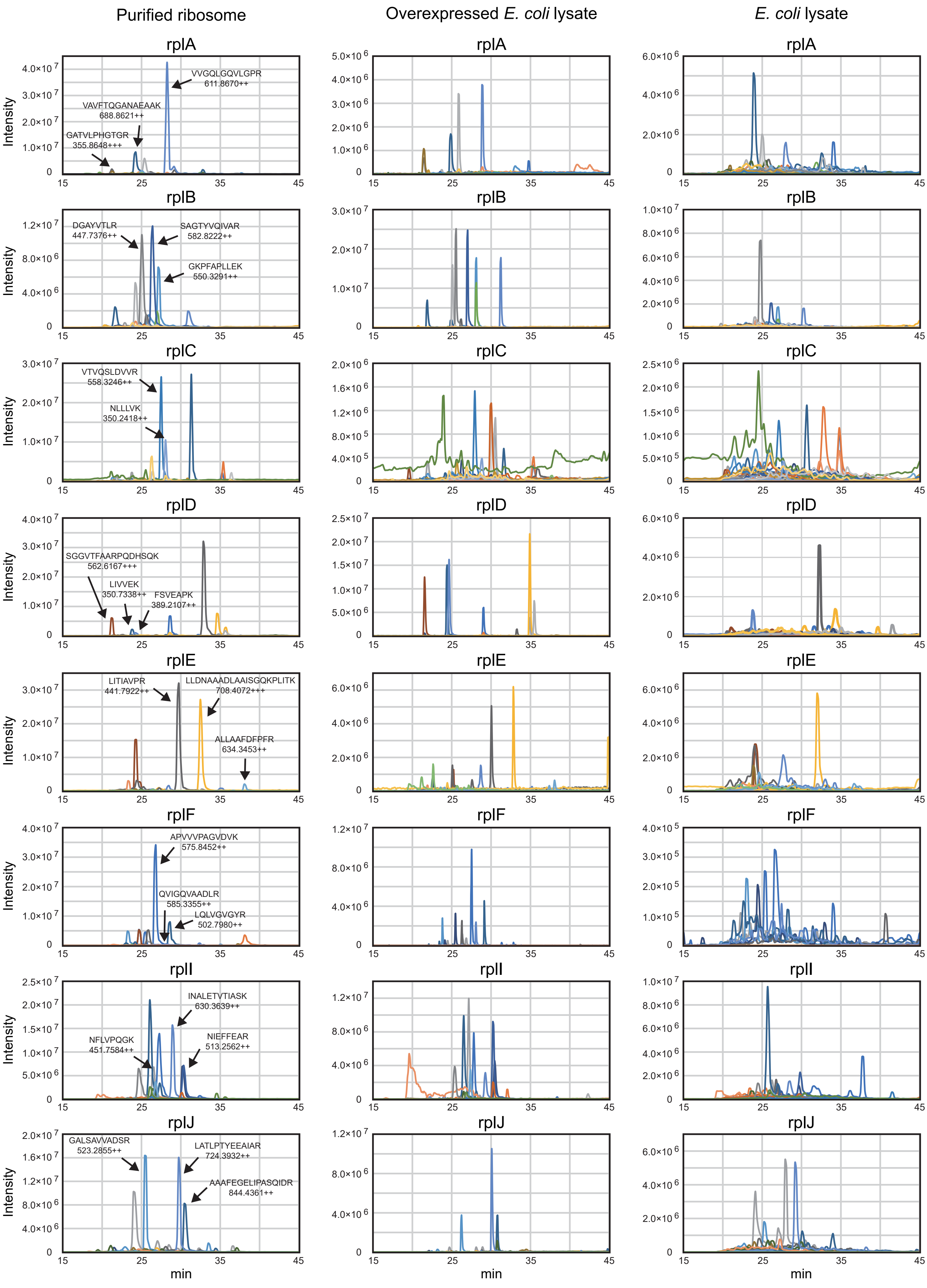

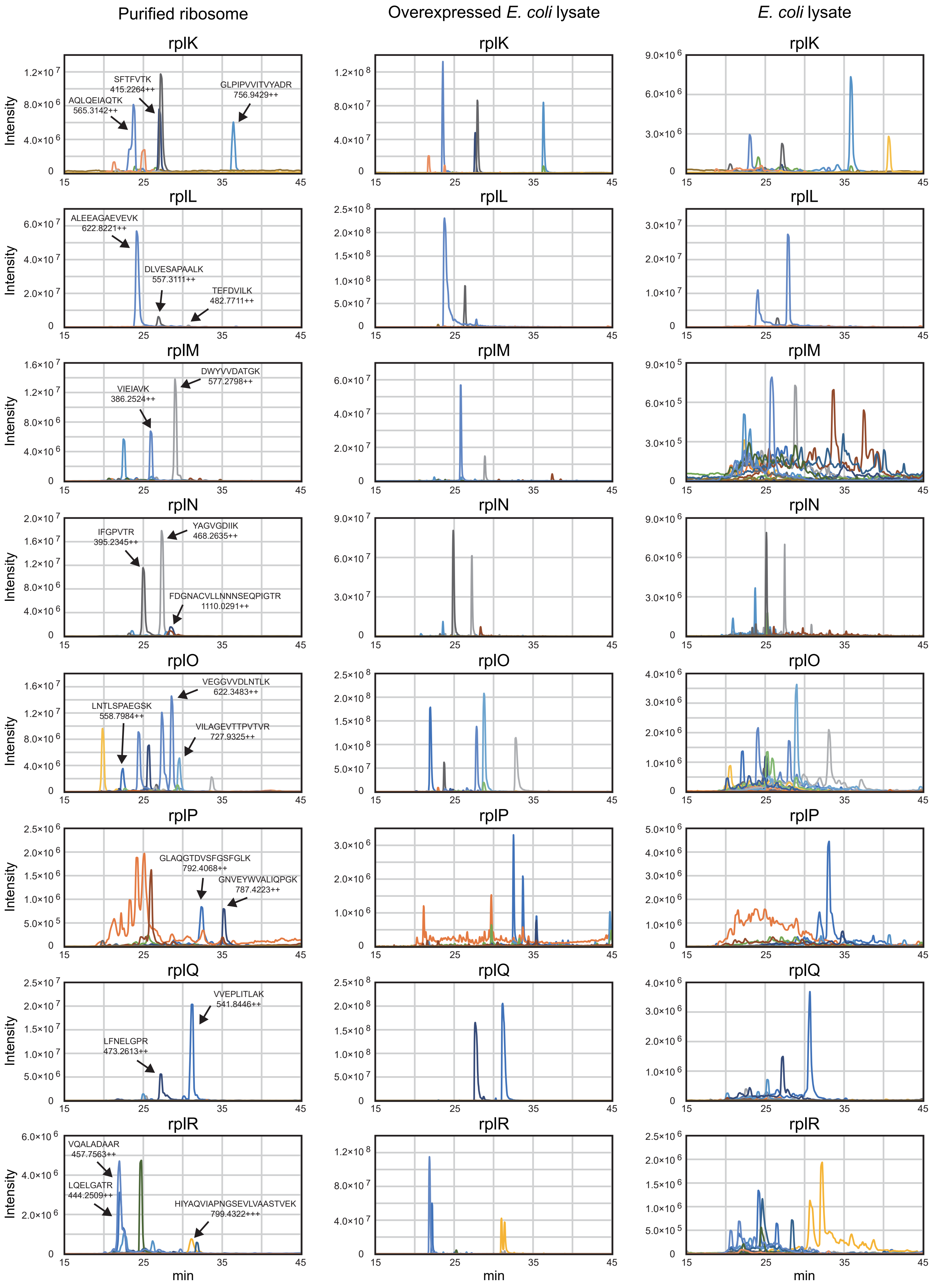
Purified ribosome
Overexpressed E. coli lysate
E. coli lysate
rplK
SFTFVTK 415.2264++
AQLQEIAQTK 565.3142++
GLPIPVVITVYADR 756.9429++
rplL
ALEEAGAEVEVK 622.8221++
DLVESAPAALK 557.3111++
TEFDVILK 482.7711++
rplM
DWYVVDATGK 577.2798++
VIEIAVK 386.2524++
rplN
YAGVGDIIK 468.2635++
IFGPVTR 395.2345++
FDGNACVLLNNNSEQPIGTR 1110.0291++
rplO
VEGGVVDLNTLK 622.3483++
LNTLSPAEGSK 558.7984++
VILAGEVTTPVTVR 727.9325++
rplP
GLAQGTDVSFGSFGLK 792.4068++
GNVEYWVALIQPGK 787.4223++
rplQ
VVEPLITLAK 541.8446++
LFNELGPR 473.2613++
rplR
VQALADAAR 457.7563++
LQELGATR 444.2509++
HIYAQVIAPNGSEVLVAASTVEK 799.4322+++
Intensity
min

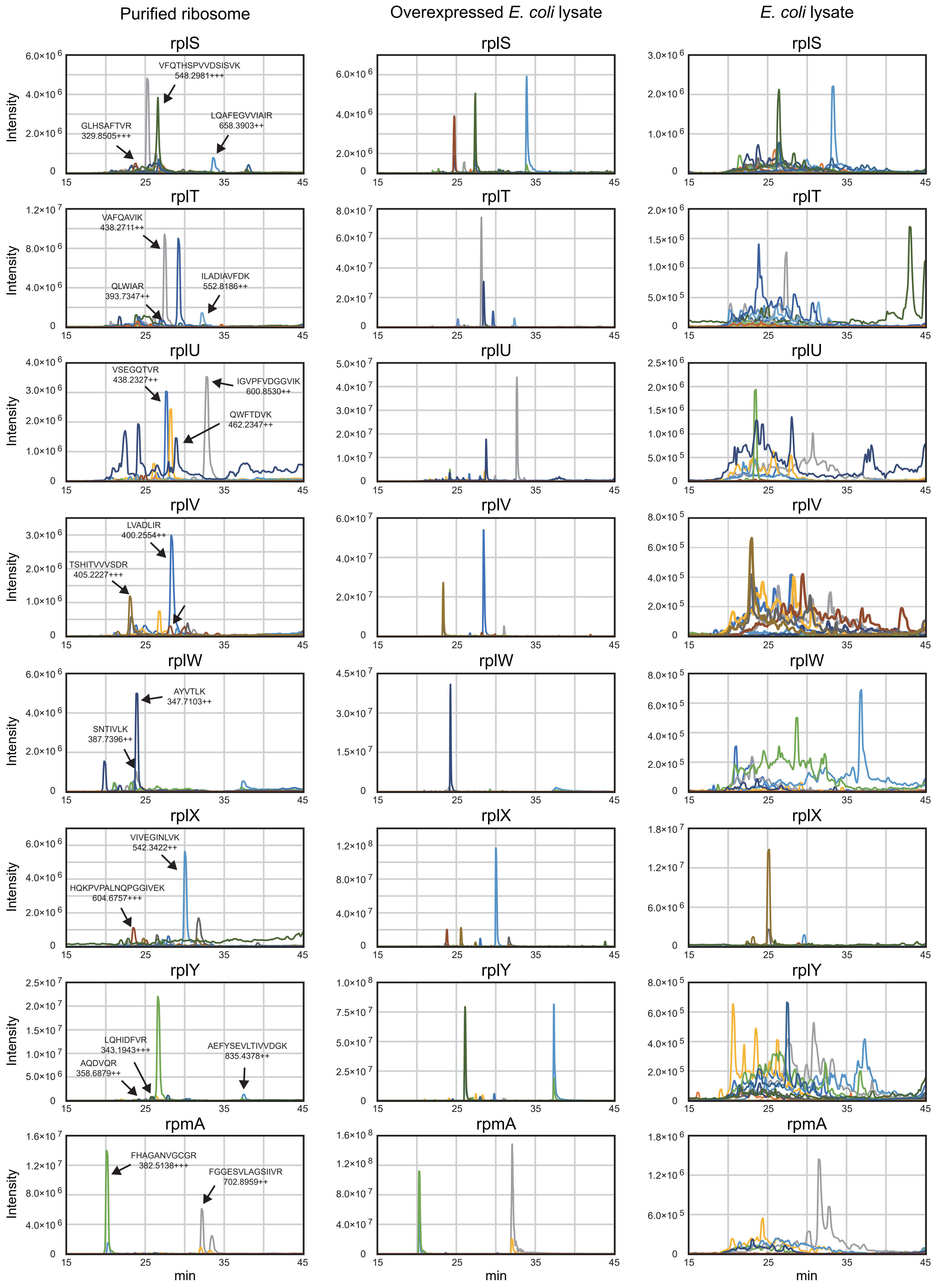

Purified ribosome
Overexpressed E. coli lysate
E. coli lysate
rplS
VFQTHSPVVDSISVK
548.2981+++
GLHSAFTVR
329.8505+++
LQAFEGVVIAIR
658.3903++
rplT
VAFQAVIK
438.2711++
QLWIAR
393.7347++
ILADIAVFDK
552.8186++
rplU
VSEGQTVR
438.2327++
IGVPFVDGGVIK
600.8530++
QWFTDVK
462.2347++
rplV
LVADLIR
400.2554++
TSHITVVVSDR
405.2227+++
rplW
AYVTLK
347.7103++
SNTIVLK
387.7396++
rplX
VIVEGINLVK
542.3422++
HQKPVPALNQPGGIVEK
604.6757+++
rplY
LQHIDFVR
343.1943+++
AQDVQR
358.6879++
AEFYSEVLTIVVDGK
835.4378++
rpmA
FHAGANVGCGR
382.5138+++
FGGESVLAGSIIVR
702.8959++
Intensity
min

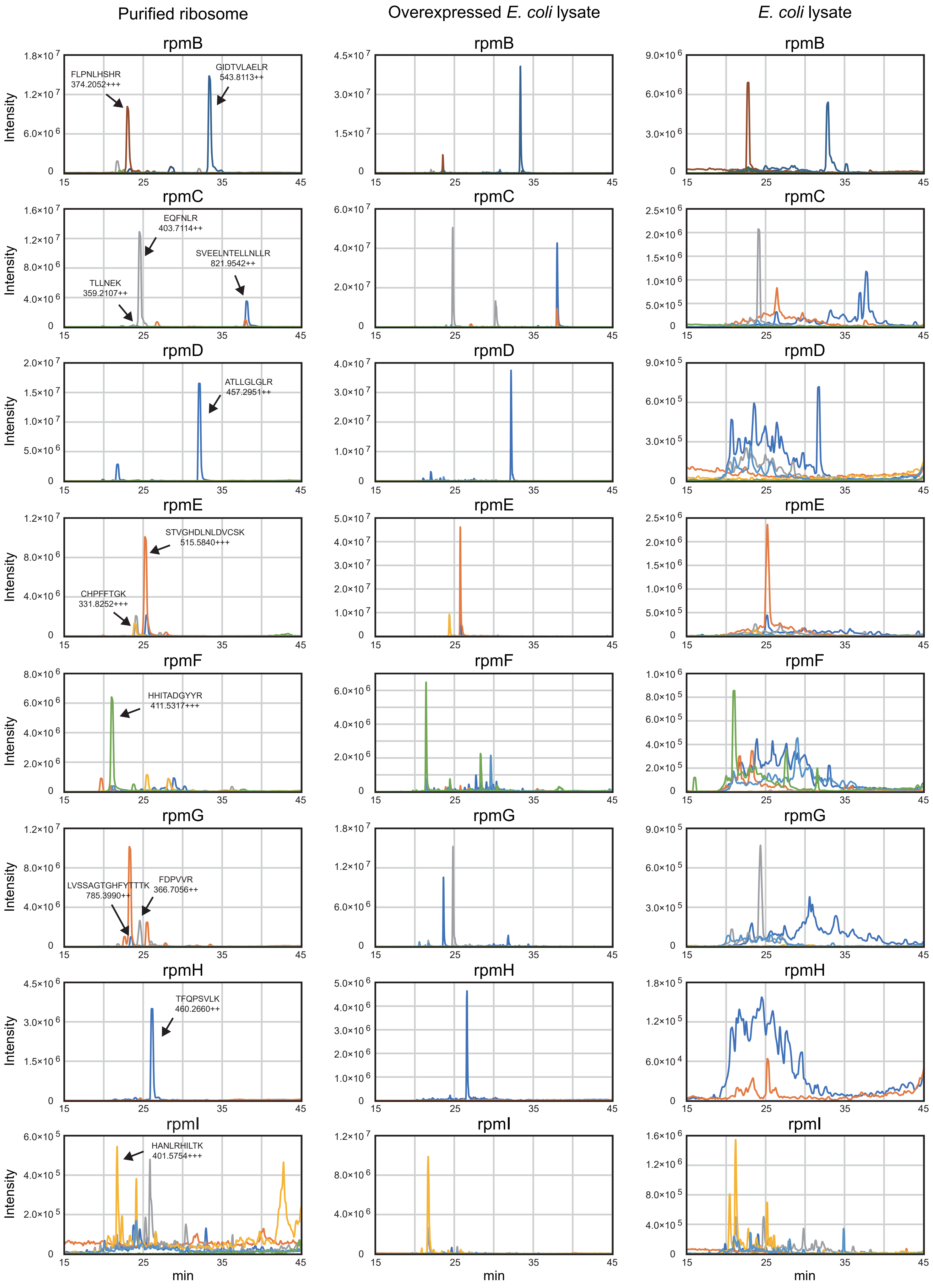

Purified ribosome
Overexpressed E. coli lysate
E. coli lysate
rpmB
FLPNLHSHR
374.2052+++
GIDTVLAELR
543.8113++
rpmC
EQFNLR
403.7114++
SVEELNTELLNLLR
821.9542++
TLLNEK
359.2107++
rpmD
ATLLGLGLR
457.2951++
rpmE
STVGHDLNLDVCSK
515.5840+++
CHPFFTGK
331.8252+++
rpmF
HHITADGYYR
411.5317+++
rpmG
FDPVVR
366.7056++
LVSSAGTGHFYTTTK
785.3990++
rpmH
TFQPSVLK
460.2660++
rpmI
HANLRHILTK
401.5754+++
Intensity
min

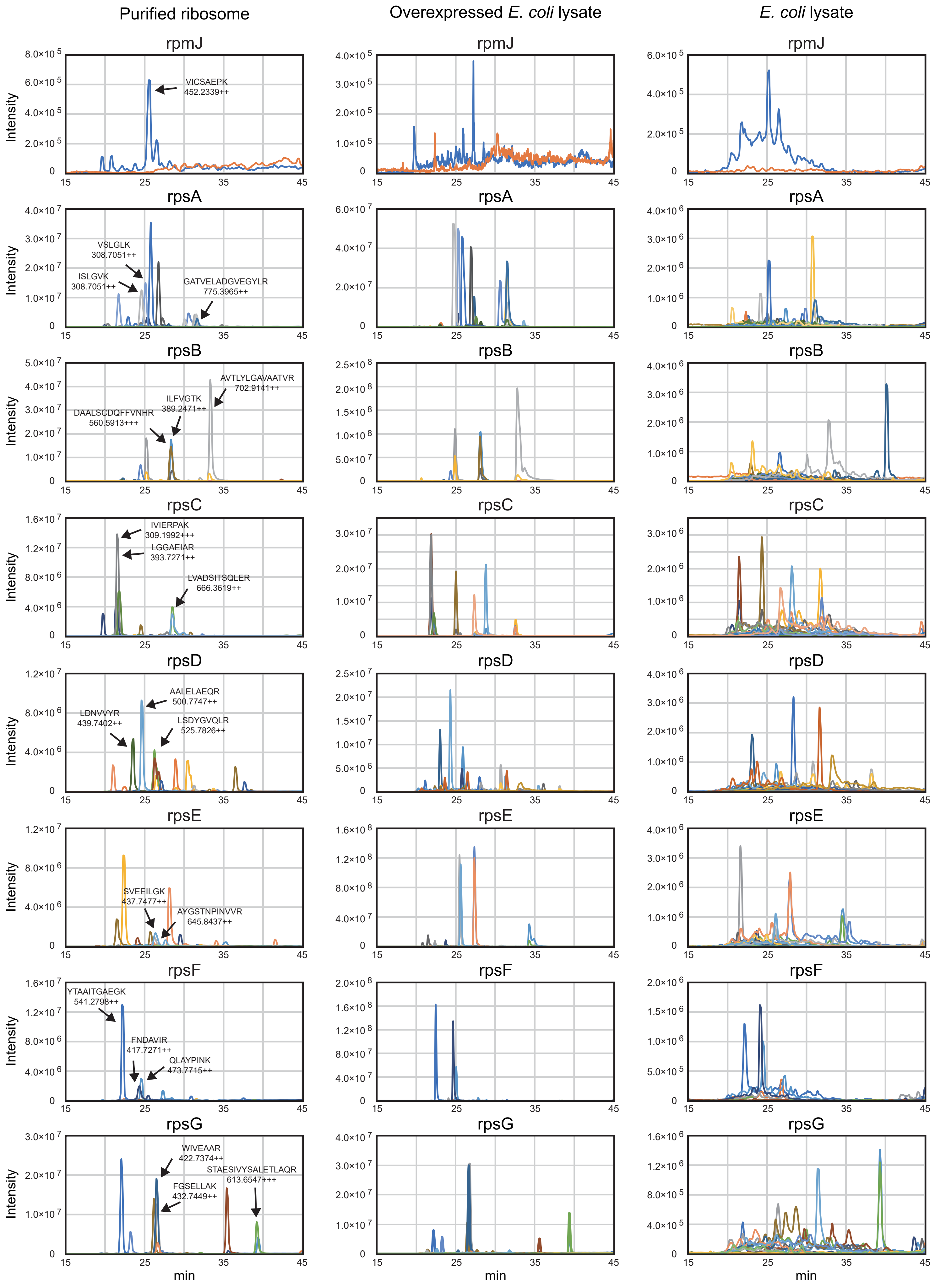

Purified ribosome
Overexpressed E. coli lysate
E. coli lysate
rpmJ
VICSAEPK
452.2339++
rpsA
VSLGLK
308.7051++
ISLGVK
308.7051++
GATVELADGVEGYLR
775.3965++
rpsB
AVTLYLGAVAATVR
702.9141++
ILFVGTK
389.2471++
DAALSCDQFFVNHR
560.5913+++
rpsC
IVIERPAK
309.1992+++
LGGAEIAR
393.7271++
LVADSITSQLER
666.3619++
rpsD
AALELAEQR
500.7747++
LDNVVYR
439.7402++
LSDYGVQLR
525.7826++
rpsE
SVEEILGK
437.7477++
AYGSTNPINVVR
645.8437++
rpsF
YTAAITGAEGK
541.2798++
FNDAVIR
417.7271++
QLAYPINK
473.7715++
rpsG
WIVEAAR
422.7374++
STAESIVYSALETLAQR
613.6547+++
FGSELLAK
432.7449++
Intensity
min

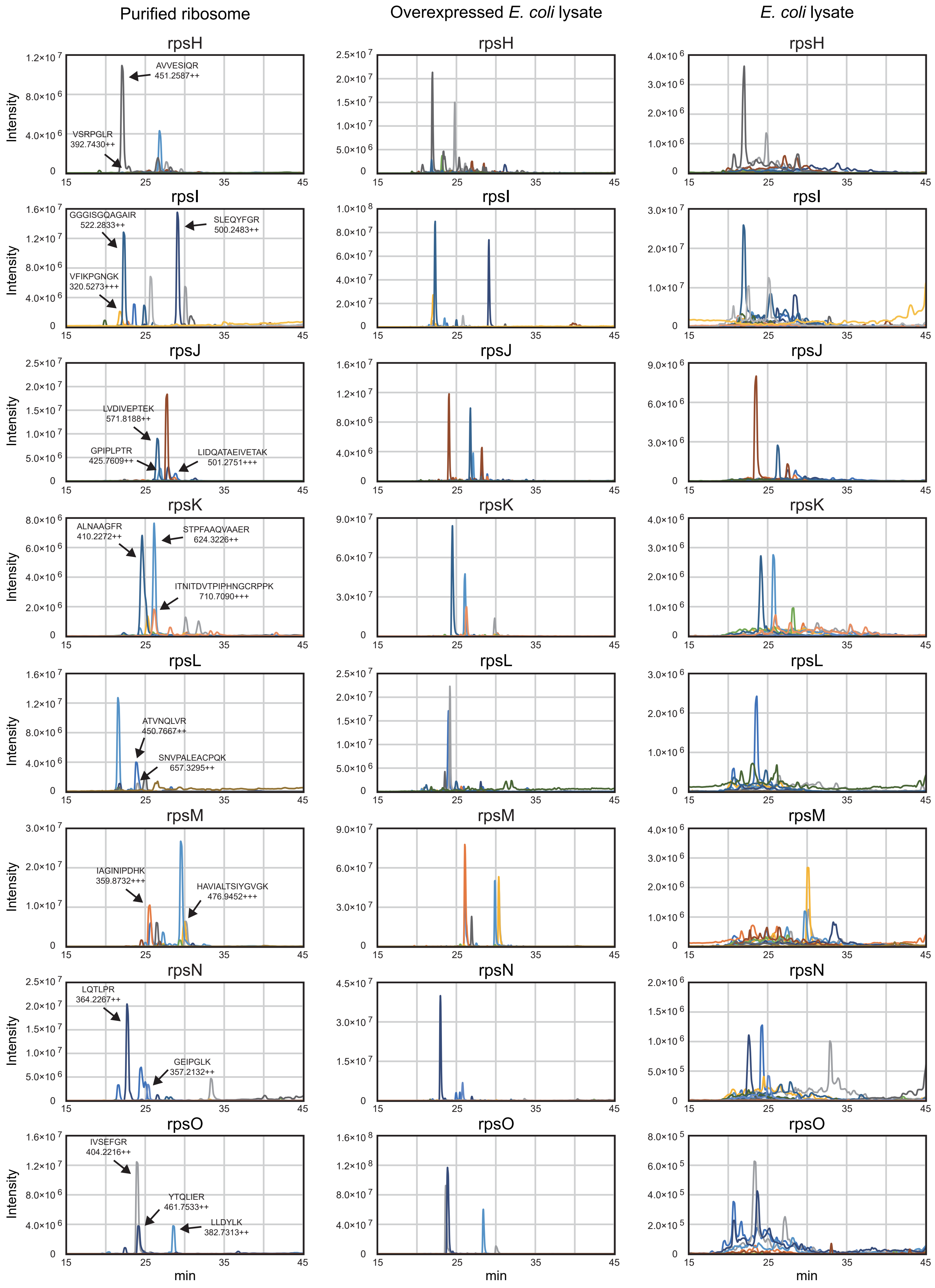

Purified ribosome
Overexpressed E. coli lysate
E. coli lysate
rpsH
AVVESIQR
451.2587++
VSRPGLR
392.7430++
rpsI
GGGISGQAGAIR
522.2833++
SLEQYFGR
500.2483++
VFIKPGNGK
320.5273+++
rpsJ
LVDIVEPTEK
571.8188++
GPIPLPTR
425.7609++
LIDQATAEIVETAK
501.2751+++
rpsK
ALNAAGFR
410.2272++
STPFAAQVAAER
624.3226++
ITNITDVTPIPHNGCRPPK
710.7090+++
rpsL
ATVNQLVR
450.7667++
SNVPALEACPQK
657.3295++
rpsM
IAGINIPDHK
359.8732+++
HAVIALTSIYGVGK
476.9452+++
rpsN
LQTLPR
364.2267++
GEIPGLK
357.2132++
rpsO
IVSEFGR
404.2216++
YTQLIER
461.7533++
LLDYLK
382.7313++
Intensity
min

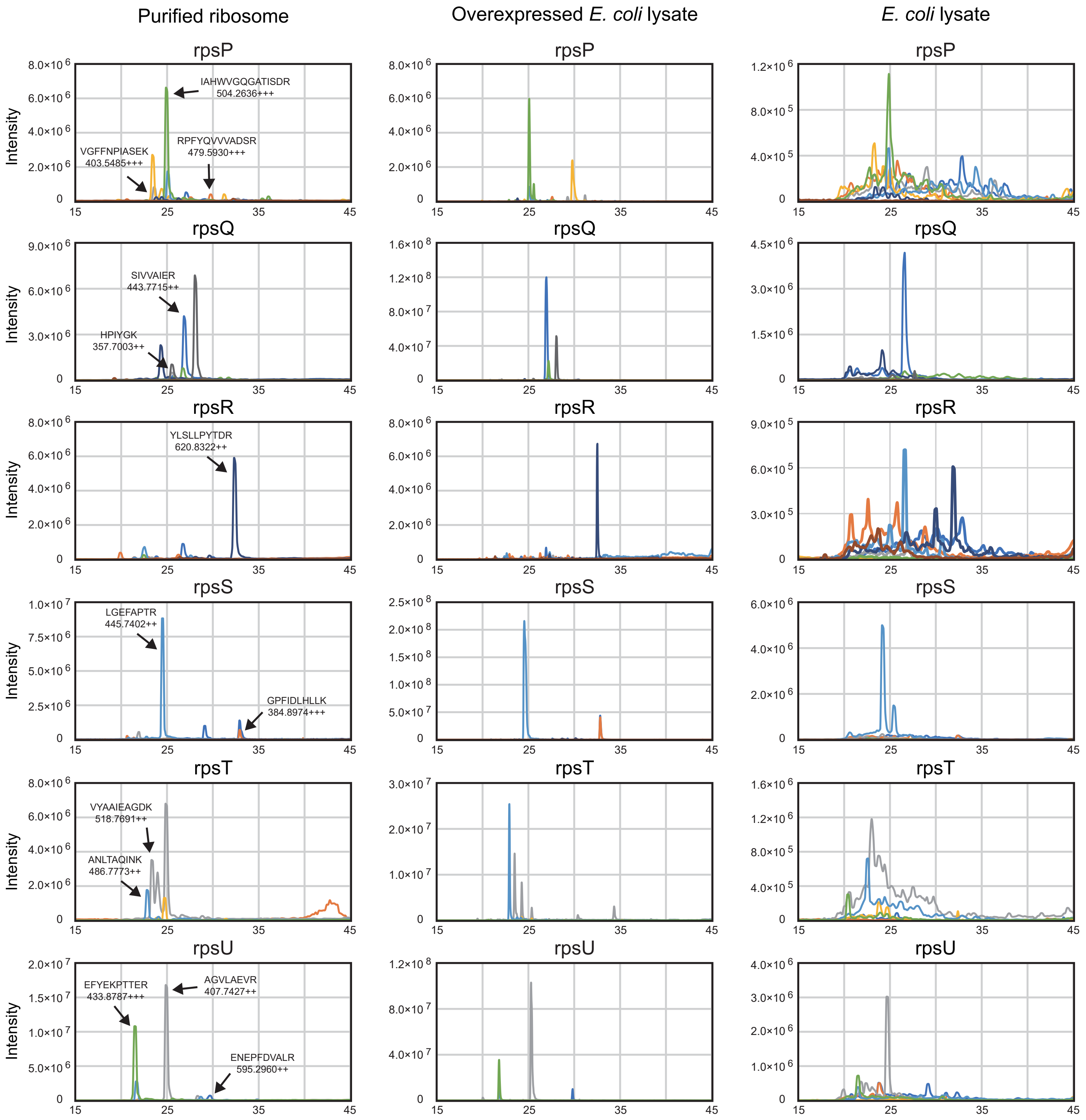

Purified ribosome
Overexpressed E. coli lysate
E. coli lysate
rpsP
IAHWVGQGATISDR
504.2636+++
VGFFNPIASEK
403.5485+++
RPFYQVVVADSR
479.5930+++
rpsQ
SIVVAIER
443.7715++
HPIYGK
357.7003++
rpsR
YLSLLPYTDR
620.8322++
rpsS
LGEFAPTR
445.7402++
GPFIDLHLLK
384.8974+++
rpsT
VYAAIEAGDK
518.7691++
ANLTAQINK
486.7773++
rpsU
EFYEKPTTER
433.8787+++
AGVLAEVR
407.7427++
ENEPFDVALR
595.2960++
Intensity
