# Supplementary material for: Selected reaction monitoring for the quantification of Escherichia coli ribosomal proteins
Source: PLoS One. 2020 Dec 14;15(12):e0236850. doi: 10.1371/journal.pone.0236850 (PMC7735604; doi:10.1371/journal.pone.0236850)
Supplement: S2 Fig — For each peptide, several transitions with intense peaks were selected based on the quantification of r-proteins from purified ribosomes. The calibration curves of all transitions are described in S3 Table. The whole peaks of all transition were provided in S1 Table. (PDF) [file pone.0236850.s002.pdf]

S2 Fig. Ion chromatograms corresponding to the selected transitions associated with peptides from ribosomal proteins.
For each peptide, several transitions with intense peaks were selected based on the quantification of r-proteins from purified ribosomes.
The calibration curves of all transitions are described in Table S2.

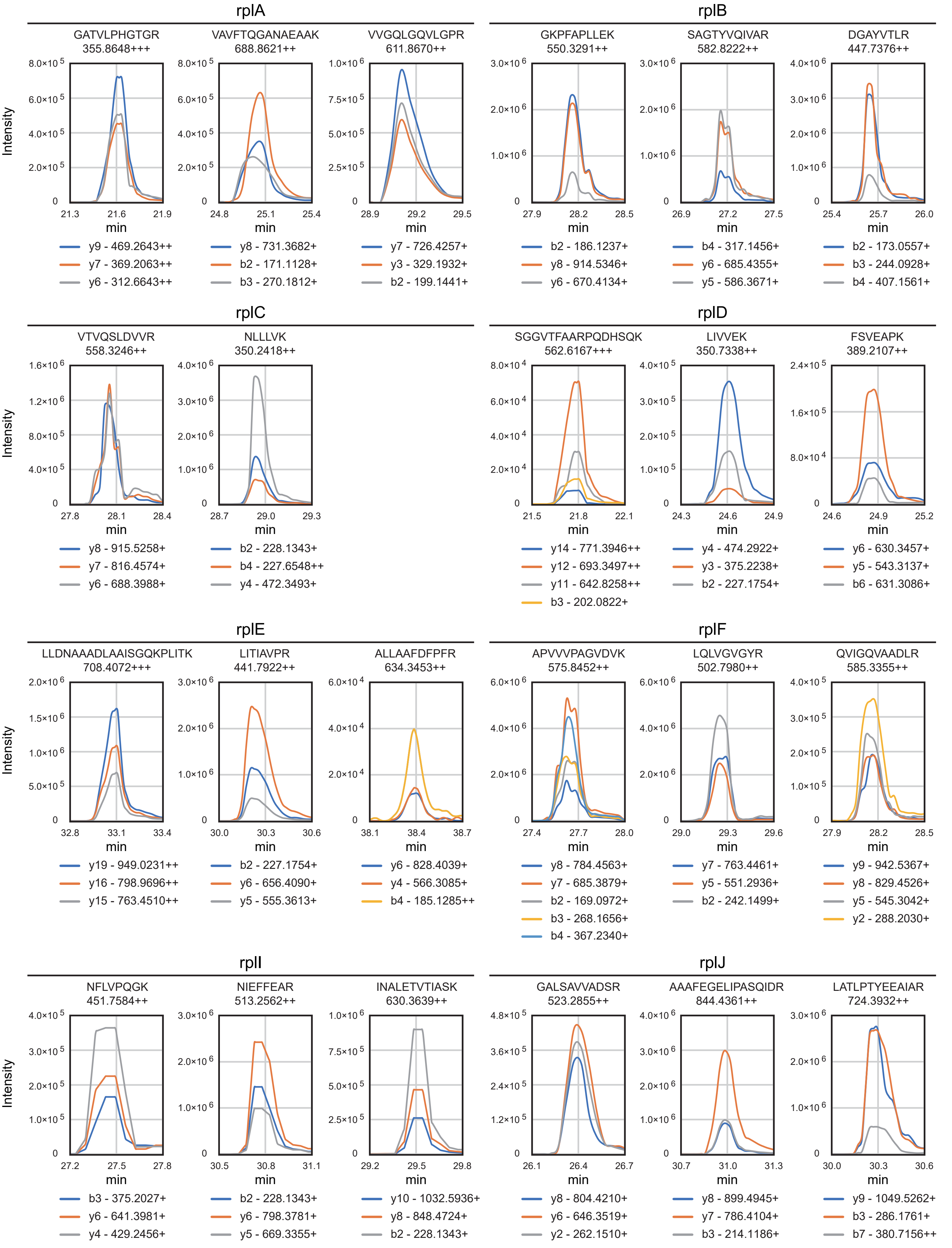

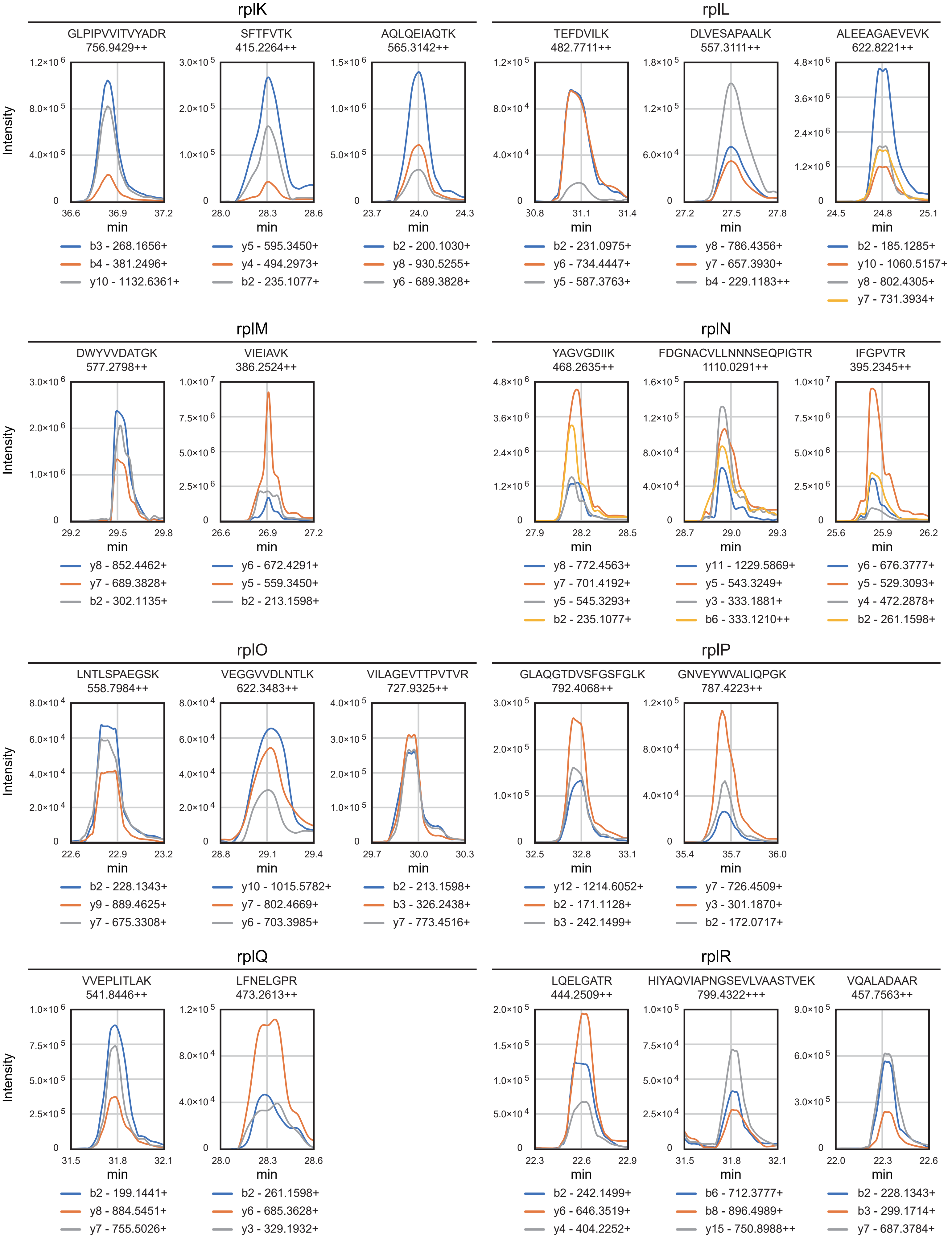
rplK
GLPIPVVITVYADR
756.9429++
SFTFVTK
415.2264++
AQLQEIAQTK
565.3142++
rplL
TEFDVILK
482.7711++
DLVESAPAALK
557.3111++
ALEEAGAEVEVK
622.8221++
Intensity
min
b3 - 268.1656+
b4 - 381.2496+
y10 - 1132.6361+
y5 - 595.3450+
y4 - 494.2973+
b2 - 235.1077+
b2 - 200.1030+
y8 - 930.5255+
y6 - 689.3828+
b2 - 231.0975+
y6 - 734.4447+
y5 - 587.3763+
y8 - 786.4356+
y7 - 657.3930+
b4 - 229.1183++
b2 - 185.1285+
y10 - 1060.5157+
y8 - 802.4305+
y7 - 731.3934+
rplM
DWYVVDATGK
577.2798++
VIEIAVK
386.2524++
rplN
YAGVGDIIK
468.2635++
FDGNACVLLNNNSEQPIGTR
1110.0291++
IFGPVTR
395.2345++
y8 - 852.4462+
y7 - 689.3828+
b2 - 302.1135+
y6 - 672.4291+
y5 - 559.3450+
b2 - 213.1598+
y8 - 772.4563+
y7 - 701.4192+
y5 - 545.3293+
b2 - 235.1077+
y11 - 1229.5869+
y5 - 543.3249+
y3 - 333.1881+
b6 - 333.1210++
y6 - 676.3777+
y5 - 529.3093+
y4 - 472.2878+
b2 - 261.1598+
rplO
LNTLSPAEGSK
558.7984++
VEGGVVDLNTLK
622.3483++
VILAGEVTTPVTVR
727.9325++
rplP
GLAQGTDVSFGSFGLK
792.4068++
GNVEYWVALIQPGK
787.4223++
b2 - 228.1343+
y9 - 889.4625+
y7 - 675.3308+
y10 - 1015.5782+
y7 - 802.4669+
y6 - 703.3985+
b2 - 213.1598+
b3 - 326.2438+
y7 - 773.4516+
y12 - 1214.6052+
b2 - 171.1128+
b3 - 242.1499+
y7 - 726.4509+
y3 - 301.1870+
b2 - 172.0717+
rplQ
VVEPLITLAK
541.8446++
LFNELGPR
473.2613++
rplR
LQELGATR
444.2509++
HIYAQVIAPNGSEVLVAASTVEK
799.4322+++
VQALADAAR
457.7563++
b2 - 199.1441+
y8 - 884.5451+
y7 - 755.5026+
b2 - 261.1598+
y6 - 685.3628+
y3 - 329.1932+
b2 - 242.1499+
y6 - 646.3519+
y4 - 404.2252+
b6 - 712.3777+
b8 - 896.4989+
y15 - 750.8988++
b2 - 228.1343+
b3 - 299.1714+
y7 - 687.3784+

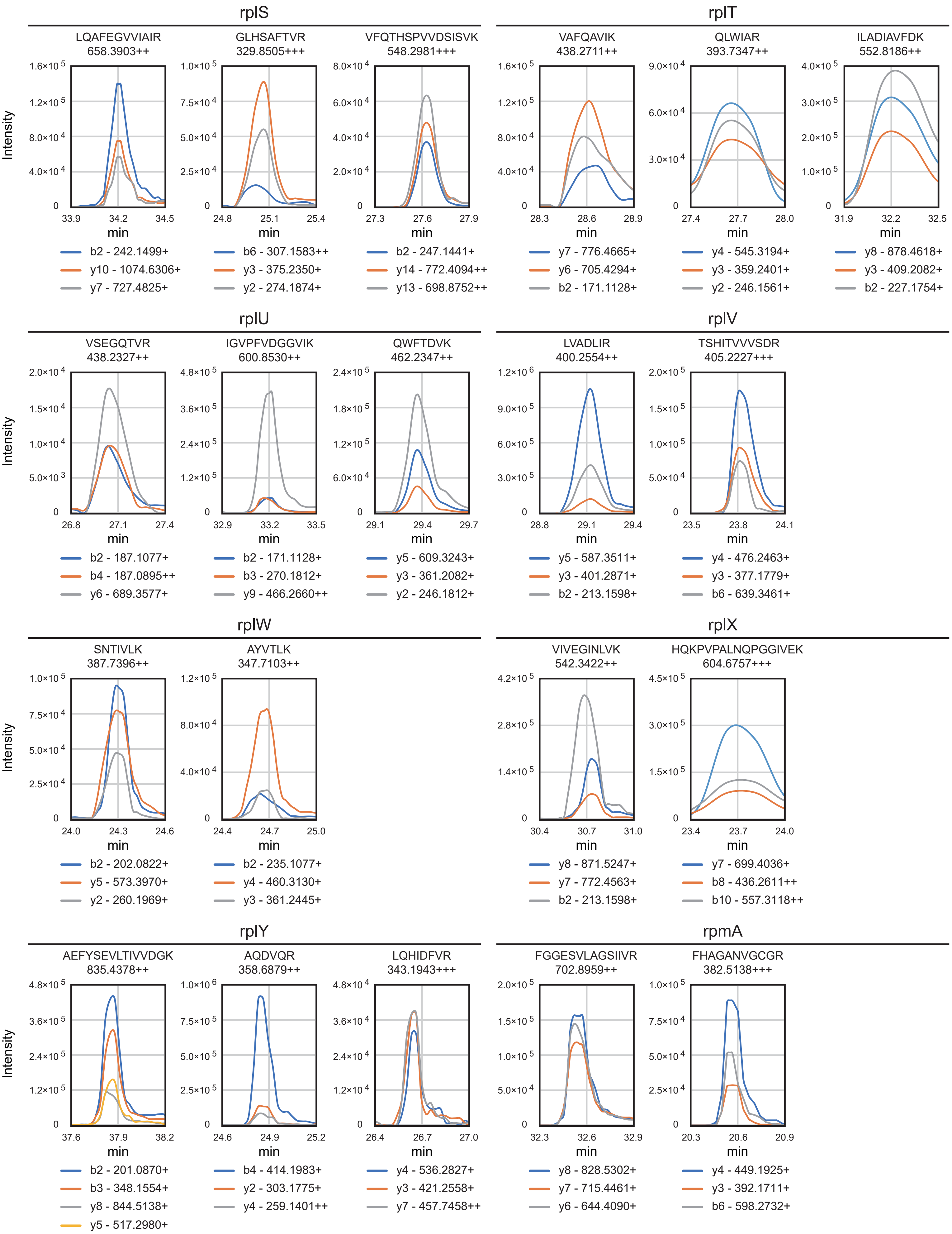
rplS
LQAFEGVVIAIR
658.3903++
b2 - 242.1499+
y10 - 1074.6306+
y7 - 727.4825+
GLHSAFTVR
329.8505+++
b6 - 307.1583++
y3 - 375.2350+
y2 - 274.1874+
VFQTHSPVVDSISVK
548.2981+++
b2 - 247.1441+
y14 - 772.4094++
y13 - 698.8752++
rplT
VAFQAVIK
438.2711++
y7 - 776.4665+
y6 - 705.4294+
b2 - 171.1128+
QLWIAR
393.7347++
y4 - 545.3194+
y3 - 359.2401+
y2 - 246.1561+
ILADIAVFDK
552.8186++
y8 - 878.4618+
y3 - 409.2082+
b2 - 227.1754+
rplU
VSEGQTVR
438.2327++
b2 - 187.1077+
b4 - 187.0895++
y6 - 689.3577+
IGVPFVDGGVIK
600.8530++
b2 - 171.1128+
b3 - 270.1812+
y9 - 466.2660++
QWFTDVK
462.2347++
y5 - 609.3243+
y3 - 361.2082+
y2 - 246.1812+
rplV
LVADLIR
400.2554++
y5 - 587.3511+
y3 - 401.2871+
b2 - 213.1598+
TSHITVVVSDR
405.2227+++
y4 - 476.2463+
y3 - 377.1779+
b6 - 639.3461+
rplW
SNTIVLK
387.7396++
b2 - 202.0822+
y5 - 573.3970+
y2 - 260.1969+
AYVTLK
347.7103++
b2 - 235.1077+
y4 - 460.3130+
y3 - 361.2445+
rplX
VIVEGINLVK
542.3422++
y8 - 871.5247+
y7 - 772.4563+
b2 - 213.1598+
HQKPVPALNQPGGIVEK
604.6757+++
y7 - 699.4036+
b8 - 436.2611++
b10 - 557.3118++
rplY
AEFYSEVLTIVVDGK
835.4378++
b2 - 201.0870+
b3 - 348.1554+
y8 - 844.5138+
y5 - 517.2980+
AQDVQR
358.6879++
b4 - 414.1983+
y2 - 303.1775+
y4 - 259.1401++
LQHIDFVR
343.1943+++
y4 - 536.2827+
y3 - 421.2558+
y7 - 457.7458++
rpmA
FGGESVLAGSIIVR
702.8959++
y8 - 828.5302+
y7 - 715.4461+
y6 - 644.4090+
FHAGANVGCGR
382.5138+++
y4 - 449.1925+
y3 - 392.1711+
b6 - 598.2732+
Intensity
min

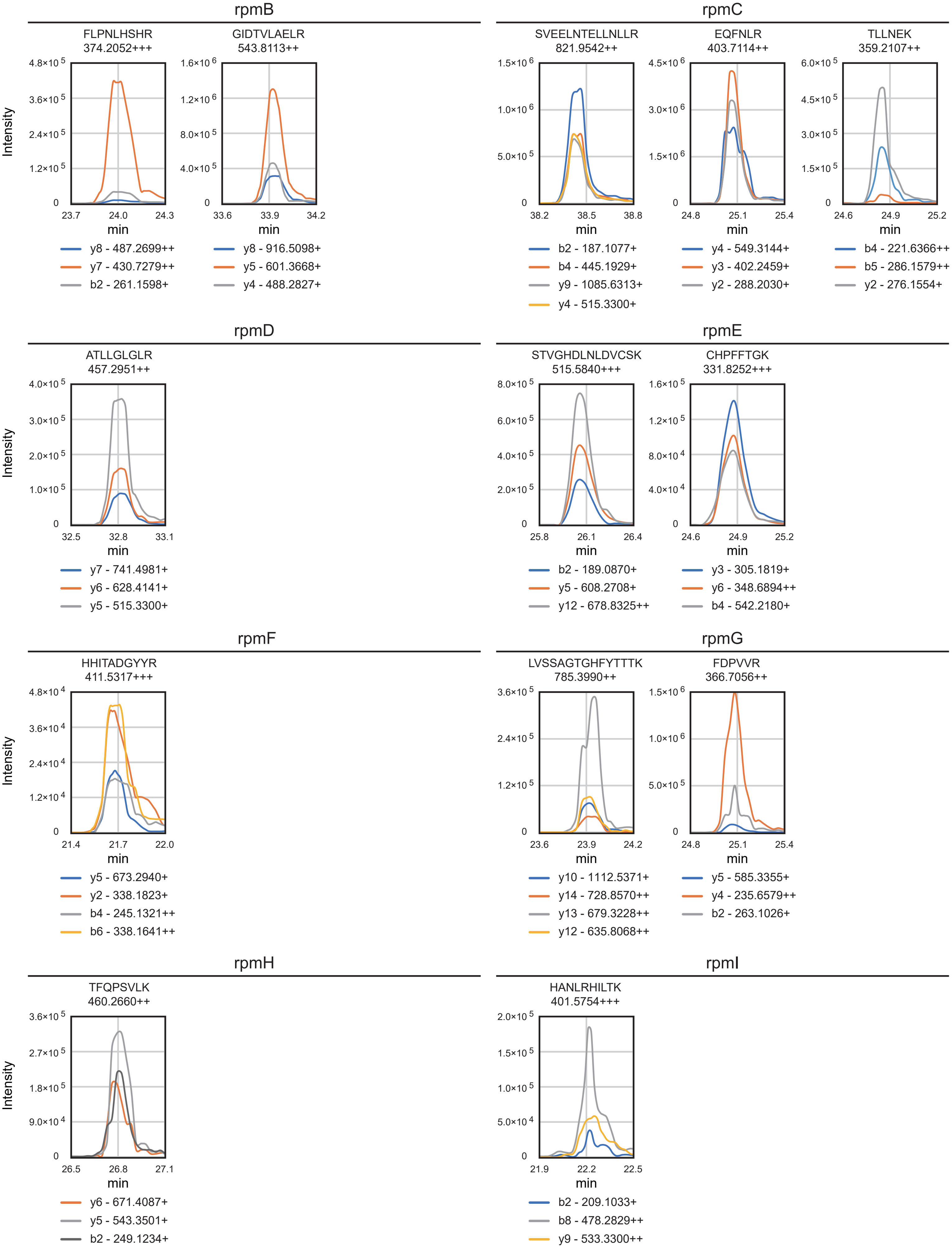

rpmB
FLPNLHSHR
374.2052+++
GIDTVLAELR
543.8113++
Intensity
4.8×10 5
3.6×10 5
2.4×10 5
1.2×10 5
0
23.7
24.0
24.3
min
1.6×10 6
1.2×10 6
8.0×10 5
4.0×10 5
33.6
33.9
34.2
y8 - 487.2699++
y7 - 430.7279++
b2 - 261.1598+
y8 - 916.5098+
y5 - 601.3668+
y4 - 488.2827+
rpmC
SVEELNTELLNLLR
821.9542++
EQFNLR
403.7114++
TLLNEK
359.2107++
1.5×10 6
1.0×10 6
5.0×10 5
38.2
38.5
38.8
4.5×10 6
3.0×10 6
1.5×10 6
24.8
25.1
25.4
6.0×10 5
4.5×10 5
3.0×10 5
1.5×10 5
24.6
24.9
25.2
b2 - 187.1077+
b4 - 445.1929+
y9 - 1085.6313+
y4 - 515.3300+
y4 - 549.3144+
y3 - 402.2459+
y2 - 288.2030+
b4 - 221.6366++
b5 - 286.1579++
y2 - 276.1554+
rpmD
ATLLGLGLR
457.2951++
4.0×10 5
3.0×10 5
2.0×10 5
1.0×10 5
32.5
32.8
33.1
y7 - 741.4981+
y6 - 628.4141+
y5 - 515.3300+
rpmE
STVGHDLNLDVCSK
515.5840+++
CHPFFTGK
331.8252+++
8.0×10 5
6.0×10 5
4.0×10 5
2.0×10 5
25.8
26.1
26.4
1.6×10 5
1.2×10 5
8.0×10 4
4.0×10 4
24.6
24.9
25.2
b2 - 189.0870+
y5 - 608.2708+
y12 - 678.8325++
y3 - 305.1819+
y6 - 348.6894++
b4 - 542.2180+
rpmF
HHITADGYYR
411.5317+++
4.8×10 4
3.6×10 4
2.4×10 4
1.2×10 4
21.4
21.7
22.0
y5 - 673.2940+
y2 - 338.1823+
b4 - 245.1321++
b6 - 338.1641++
rpmG
LVSSAGTGHFYTTTK
785.3990++
FDPVVR
366.7056++
3.6×10 5
2.4×10 5
1.2×10 5
23.6
23.9
24.2
1.5×10 6
1.0×10 6
5.0×10 5
24.8
25.1
25.4
y10 - 1112.5371+
y14 - 728.8570++
y13 - 679.3228++
y12 - 635.8068++
y5 - 585.3355+
y4 - 235.6579++
b2 - 263.1026+
rpmH
TFQPSVLK
460.2660++
3.6×10 5
2.7×10 5
1.8×10 5
9.0×10 4
26.5
26.8
27.1
y6 - 671.4087+
y5 - 543.3501+
b2 - 249.1234+
rpmI
HANLRHILTK
401.5754+++
2.0×10 5
1.5×10 5
1.0×10 5
5.0×10 4
21.9
22.2
22.5
b2 - 209.1033+
b8 - 478.2829++
y9 - 533.3300++

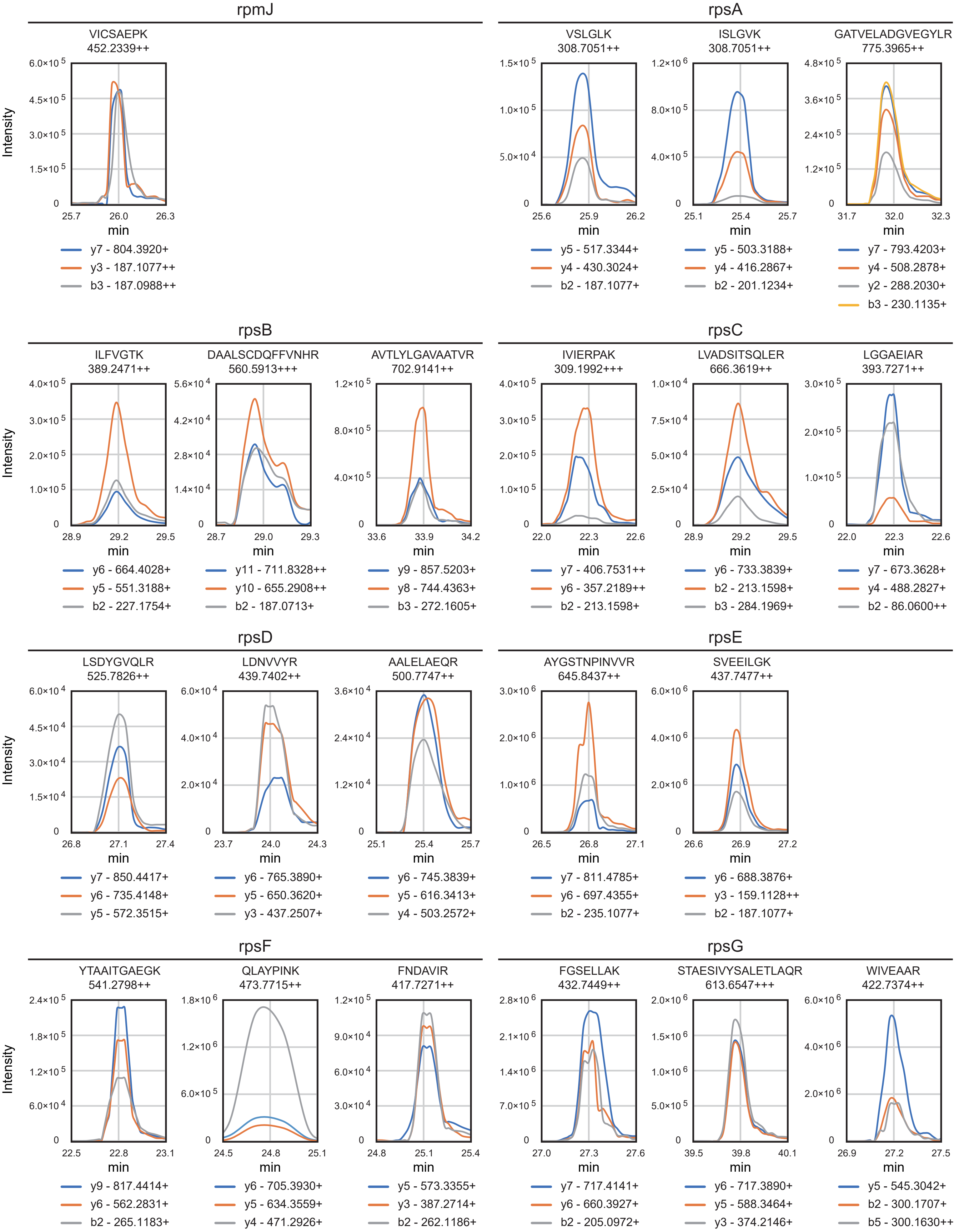

rpmJ
VICSAEPK
452.2339++
Intensity
min
y7 - 804.3920+
y3 - 187.1077++
b3 - 187.0988++
rpsA
VSLGLK
308.7051++
y5 - 517.3344+
y4 - 430.3024+
b2 - 187.1077+
ISLGVK
308.7051++
y5 - 503.3188+
y4 - 416.2867+
b2 - 201.1234+
GATVELADGVEGYLR
775.3965++
y7 - 793.4203+
y4 - 508.2878+
y2 - 288.2030+
b3 - 230.1135+
rpsB
ILFVGTK
389.2471++
y6 - 664.4028+
y5 - 551.3188+
b2 - 227.1754+
DAALSCDQFFVNHR
560.5913+++
y11 - 711.8328++
y10 - 655.2908++
b2 - 187.0713+
AVTLYLGAVAATVR
702.9141++
y9 - 857.5203+
y8 - 744.4363+
b3 - 272.1605+
rpsC
IVIERPAK
309.1992+++
y7 - 406.7531++
y6 - 357.2189++
b2 - 213.1598+
LVADSITSQLER
666.3619++
y6 - 733.3839+
b2 - 213.1598+
b3 - 284.1969+
LGGAEIAR
393.7271++
y7 - 673.3628+
y4 - 488.2827+
b2 - 86.0600++
rpsD
LSDYGVQLR
525.7826++
y7 - 850.4417+
y6 - 735.4148+
y5 - 572.3515+
LDNVVYR
439.7402++
y6 - 765.3890+
y5 - 650.3620+
y3 - 437.2507+
AALELAEQR
500.7747++
y6 - 745.3839+
y5 - 616.3413+
y4 - 503.2572+
rpsE
AYGSTNPINVVR
645.8437++
y7 - 811.4785+
y6 - 697.4355+
b2 - 235.1077+
SVEEILGK
437.7477++
y6 - 688.3876+
y3 - 159.1128++
b2 - 187.1077+
rpsF
YTAAITGAEGK
541.2798++
y9 - 817.4414+
y6 - 562.2831+
b2 - 265.1183+
QLAYPINK
473.7715++
y6 - 705.3930+
y5 - 634.3559+
y4 - 471.2926+
FNDAVIR
417.7271++
y5 - 573.3355+
y3 - 387.2714+
b2 - 262.1186+
rpsG
FGSELLAK
432.7449++
y7 - 717.4141+
y6 - 660.3927+
b2 - 205.0972+
STAESIVYSALETLAQR
613.6547+++
y6 - 717.3890+
y5 - 588.3464+
y3 - 374.2146+
WIVEAAR
422.7374++
y5 - 545.3042+
b2 - 300.1707+
b5 - 300.1630++

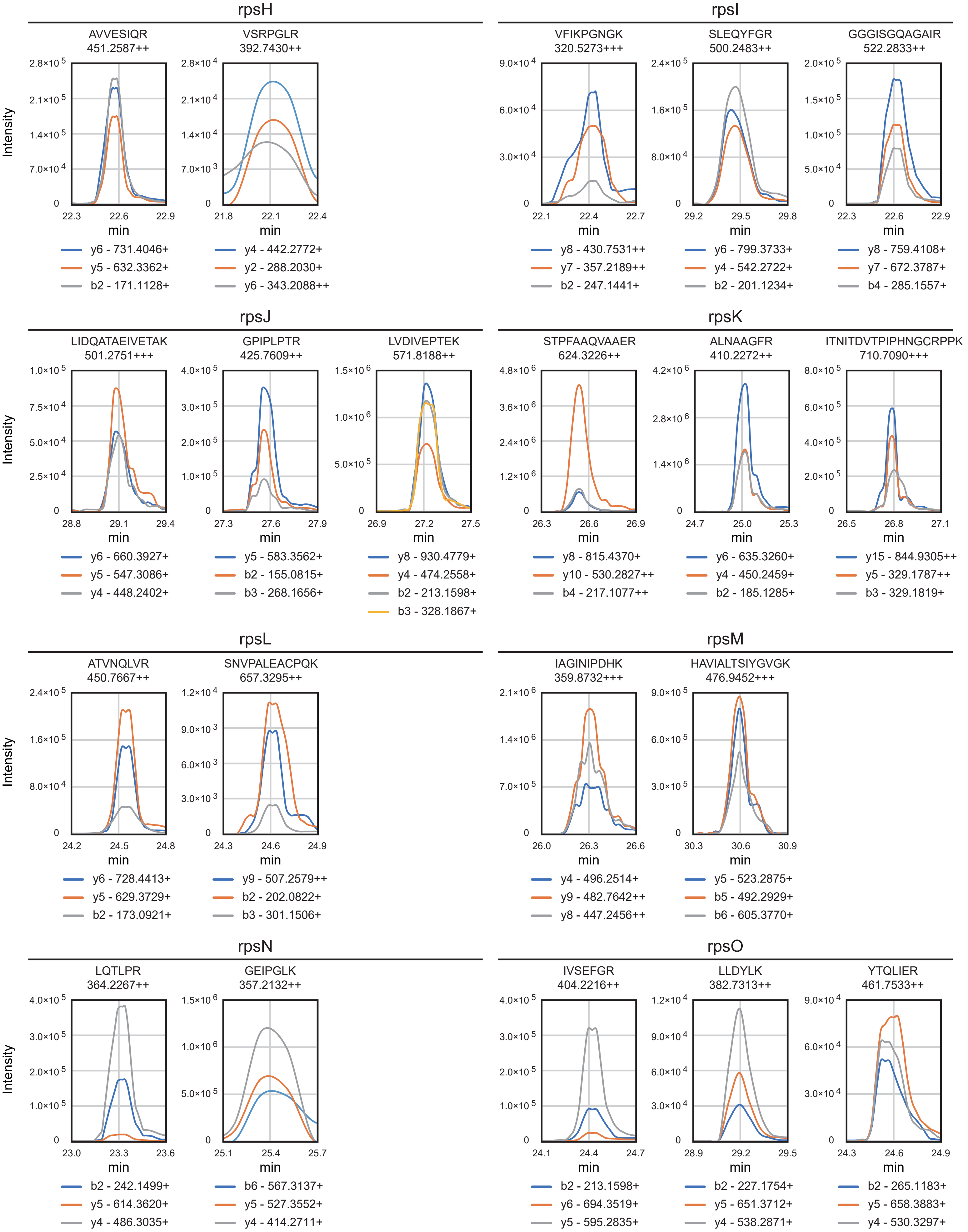
rpsH
AVVESIQR
451.2587++
y6 - 731.4046+
y5 - 632.3362+
b2 - 171.1128+
VSRPGLR
392.7430++
y4 - 442.2772+
y2 - 288.2030+
y6 - 343.2088++
rpsI
VFIKPGNGK
320.5273+++
y8 - 430.7531++
y7 - 357.2189++
b2 - 247.1441+
SLEQYFGR
500.2483++
y6 - 799.3733+
y4 - 542.2722+
b2 - 201.1234+
GGGISGQAGAIR
522.2833++
y8 - 759.4108+
y7 - 672.3787+
b4 - 285.1557+
rpsJ
LIDQATAEIVETAK
501.2751+++
y6 - 660.3927+
y5 - 547.3086+
y4 - 448.2402+
GPIPLPTR
425.7609++
y5 - 583.3562+
b2 - 155.0815+
b3 - 268.1656+
LVDIVEPTEK
571.8188++
y8 - 930.4779+
y4 - 474.2558+
b2 - 213.1598+
b3 - 328.1867+
rpsK
STPFAAQVAAER
624.3226++
y8 - 815.4370+
y10 - 530.2827++
b4 - 217.1077++
ALNAAGFR
410.2272++
y6 - 635.3260+
y4 - 450.2459+
b2 - 185.1285+
ITNITDVTPIPHNGCRPPK
710.7090+++
y15 - 844.9305++
y5 - 329.1787++
b3 - 329.1819+
rpsL
ATVNQLVR
450.7667++
y6 - 728.4413+
y5 - 629.3729+
b2 - 173.0921+
SNVPALEACPQK
657.3295++
y9 - 507.2579++
b2 - 202.0822+
b3 - 301.1506+
rpsM
IAGINIPDHK
359.8732+++
y4 - 496.2514+
y9 - 482.7642++
y8 - 447.2456++
HAVIALTSIYGVGK
476.9452+++
y5 - 523.2875+
b5 - 492.2929+
b6 - 605.3770+
rpsN
LQTLPR
364.2267++
b2 - 242.1499+
y5 - 614.3620+
y4 - 486.3035+
GEIPGLK
357.2132++
b6 - 567.3137+
y5 - 527.3552+
y4 - 414.2711+
rpsO
IVSEFGR
404.2216++
b2 - 213.1598+
y6 - 694.3519+
y5 - 595.2835+
LLDYLK
382.7313++
b2 - 227.1754+
y5 - 651.3712+
y4 - 538.2871+
YTQLIER
461.7533++
b2 - 265.1183+
y5 - 658.3883+
y4 - 530.3297+
Intensity
min

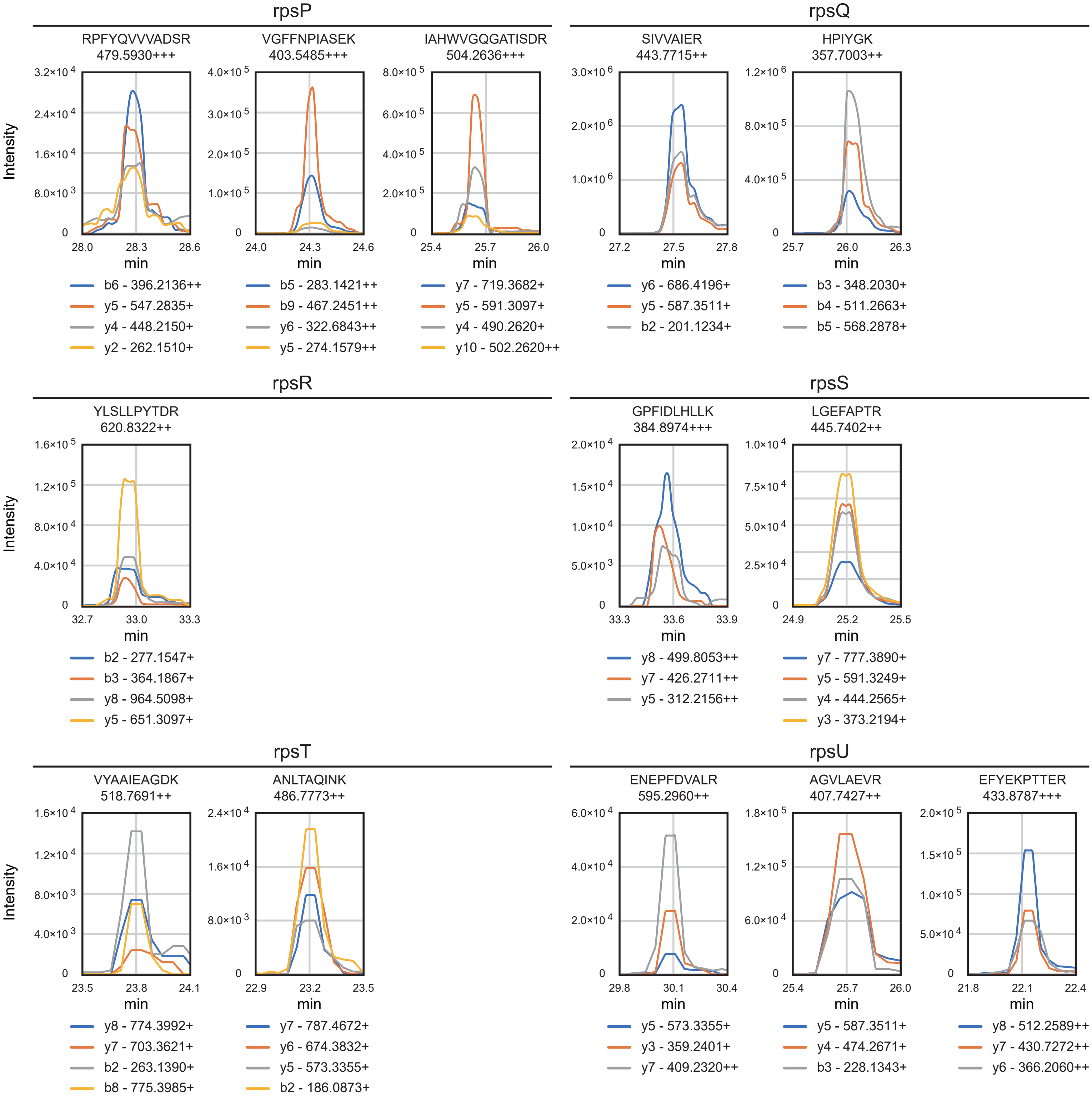

rpsP
RPFYQVVVADSR
479.5930+++
VGFFNPIASEK
403.5485+++
IAHWVGQGATISDR
504.2636+++
Intensity
min
b6 - 396.2136++
y5 - 547.2835+
y4 - 448.2150+
y2 - 262.1510+
b5 - 283.1421++
b9 - 467.2451++
y6 - 322.6843++
y5 - 274.1579++
y7 - 719.3682+
y5 - 591.3097+
y4 - 490.2620+
y10 - 502.2620++
rpsQ
SIVVAIER
443.7715++
HPIYGK
357.7003++
y6 - 686.4196+
y5 - 587.3511+
b2 - 201.1234+
b3 - 348.2030+
b4 - 511.2663+
b5 - 568.2878+
rpsR
YLSLLPYTDR
620.8322++
b2 - 277.1547+
b3 - 364.1867+
y8 - 964.5098+
y5 - 651.3097+
rpsS
GPFIDLHLLK
384.8974+++
LGEFAPTR
445.7402++
y8 - 499.8053++
y7 - 426.2711++
y5 - 312.2156++
y7 - 777.3890+
y5 - 591.3249+
y4 - 444.2565+
y3 - 373.2194+
rpsT
VYAAIEAGDK
518.7691++
ANLTAQINK
486.7773++
y8 - 774.3992+
y7 - 703.3621+
b2 - 263.1390+
b8 - 775.3985+
y7 - 787.4672+
y6 - 674.3832+
y5 - 573.3355+
b2 - 186.0873+
rpsU
ENEPFDVALR
595.2960++
AGVLAEVR
407.7427++
EFYEKPTTER
433.8787+++
y5 - 573.3355+
y3 - 359.2401+
y7 - 409.2320++
y5 - 587.3511+
y4 - 474.2671+
b3 - 228.1343+
y8 - 512.2589++
y7 - 430.7272++
y6 - 366.2060++
