# Supplementary figures and images for: Selected reaction monitoring for the quantification of Escherichia coli ribosomal proteins
Source: PLoS One. 2020 Dec 14;15(12):e0236850. doi: 10.1371/journal.pone.0236850 (PMC7735604; doi:10.1371/journal.pone.0236850)

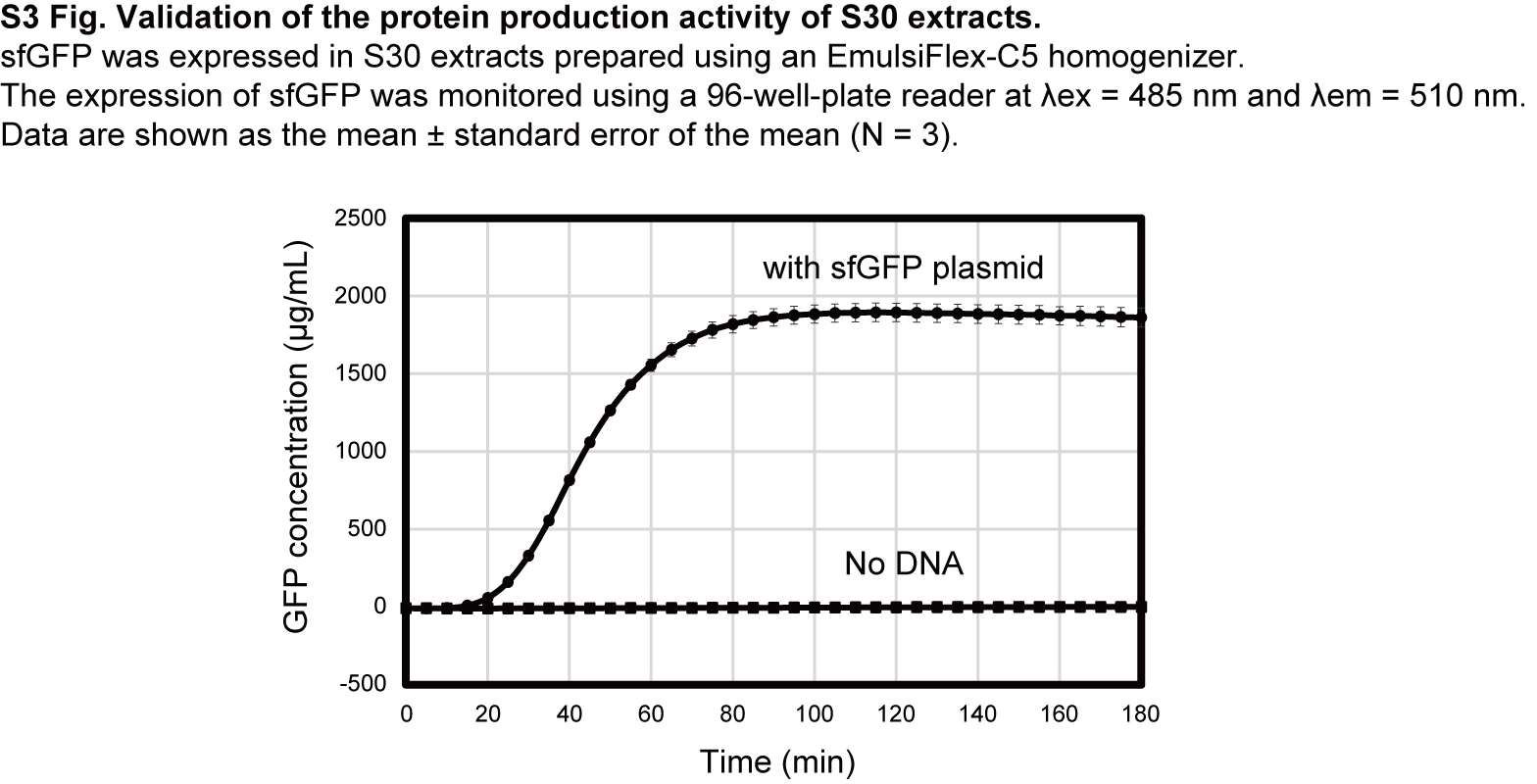

Supplement: S3 Fig — sfGFP was expressed in S30 extracts prepared using an EmulsiFlex-C5 homogenizer. The expression of sfGFP was monitored using a 96-well-plate reader at λex = 485 nm and λem = 510 nm. Data are shown as the mean ± standard error of the mean (N = 3). (TIF) [file pone.0236850.s003.tif]

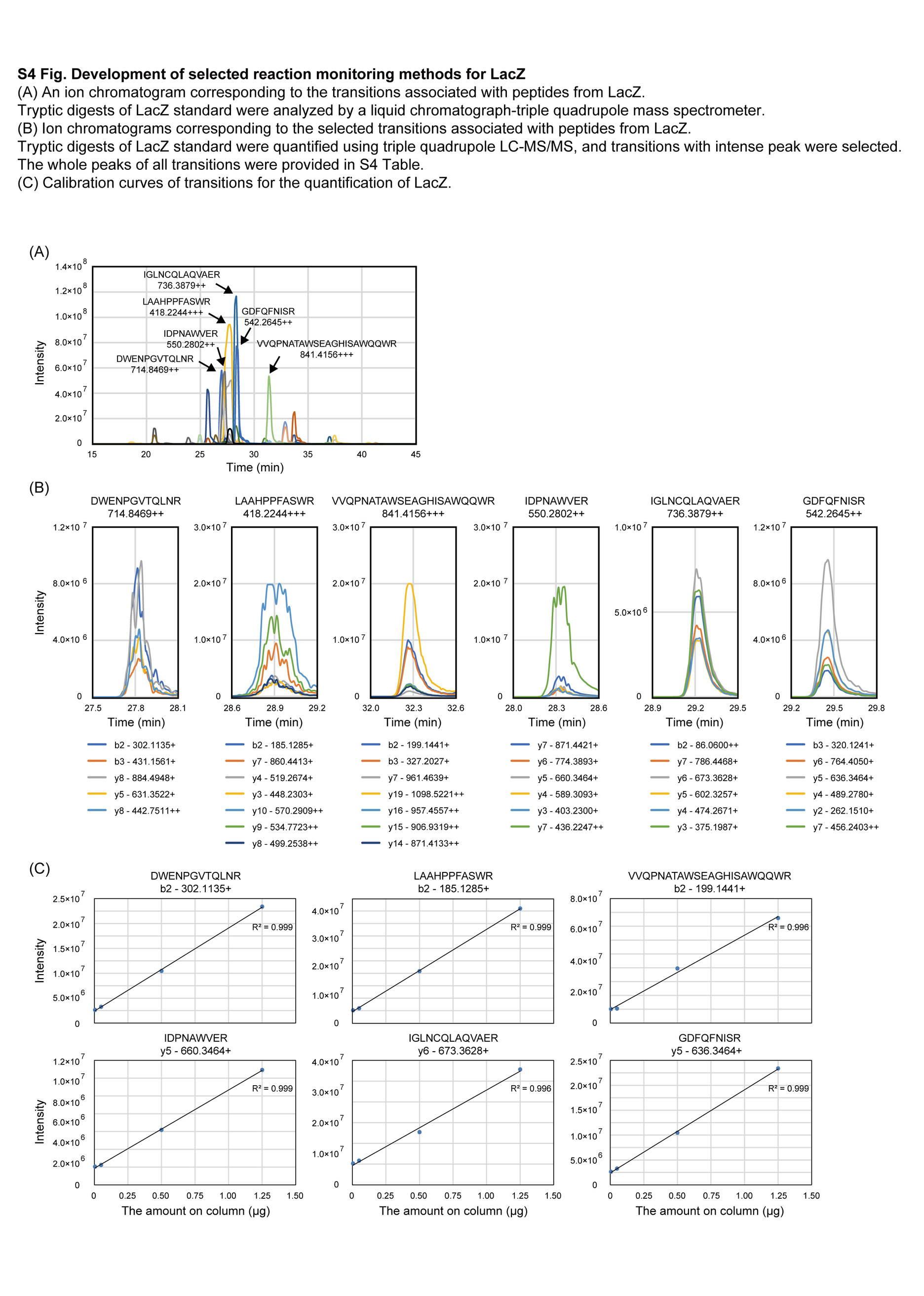

Supplement: S4 Fig — (A) An ion chromatogram corresponding to the transitions associated with peptides from LacZ. Tryptic digests of LacZ standard were analyzed by a liquid chromatograph-triple quadrupole mass spectrometer. (B) Ion chromatograms corresponding to the selected transitions associated with peptides from LacZ. Tryptic digests of LacZ standard were quantified using triple quadrupole LC-MS/MS, and transitions with intense peak were selected. The whole peaks of all transition were provided in S4 Table. (C) Calibration curves of transitions for the quantification of LacZ. (TIF) [file pone.0236850.s004.tif]
