# Supplementary material for: Selected reaction monitoring for the quantification of Escherichia coli ribosomal proteins
Source: PLoS One. 2020 Dec 14;15(12):e0236850. doi: 10.1371/journal.pone.0236850 (PMC7735604; doi:10.1371/journal.pone.0236850)
Supplement: S1 File — (PDF) [file pone.0236850.s011.pdf]

pET41a_T7_sfGFP (5735 bp)

tggcgaatgggacgcgccctgtagcggcgcattaagcgcggcgggtgtggtggttacgcgcagcgtgaccgctacacttgccagcgccctagcgcccgctcctttcg
accgcttaccctgcgcgggacatcgccgcgtaattcgcgccgcccacaccaccaatgcgcgtcgcactggcgatgtgaacggtcgcgggatcgcgggcgaggaaagc
f1 ori
20 40 60 80 100

NaeI
NgoMIV
ctttcttcccttcctttctcgccacgttcgccggctttccccgtcaagctctaaatcgggggctccctttagggttccgatttagtgctttacggcacctcgacccc
gaaagaagggaaggaaagagcggtgcaagcggccgaaaggggcagttcgagatttagcccccgagggaaatcccaaggctaaatcacgaaatgccgtggagctgggg
f1 ori
120 140 160 180 200

DraIII
BsaAI
AloI
BsaXI
aaaaaacttgattagggtgatggttcacgtagtgggccatcgccctgatagacggtttttcgccctttgacgttggagtccacgttctttaatagtggactcttgtt
tttttgaactaatcccactaccaagtgcatcacccggtagcgggactatctgccaaaaagcgggaaactgcaacctcaggtgcaagaaattatcacctgagaacaa
f1 ori
220 240 260 280 300 320

PsiI
ccaaactggaacaacactcaaccctatctcggtctattcttttgatttataagggattttgccgatttcggcctattggttaaaaaatgagctgatttaacaaaaat
ggtttgaccttgttgtgagttgggatagagccagataagaaaactaaatattccctaaaacggctaaagccggataaccaattttttactcgactaaattgtttttta
f1 ori
340 360 380 400 420

SspI
ttaacgcgaattttaacaaaatattaacgtttacaatttcaggtggcacttttcggggaaatgtgcgcggaacccctatttgtttatttttctaaatacattcaaat
aattgcgcttaaaattgttttataattgcaaatgttaaagtccaccgtgaaaagcccctttacacgcgccttggggataaacaaataaaaagatttatgtaagttta
f1 ori
440 460 480 500 520

XmnI
atgtatccgctcatgaattaattcttagaaaaactcatcgagcatcaaatgaaactgcaatttattcatatcaggattatcaataccatatttttgaaaaagccgtt
tacataggcgagtacttaattaagaatctttttgagtagctcgtagtttactttgacgttaaataagtatagtcctaatagttatggtataaaaactttttcggcaa
KanR
540 560 580 600 620 640

PflMI

tctgtaatgaaggagaaaactcaccgaggcagttccataggatggcaagatcctggtatcggtctgcgattccgactcgtccaacatcaatacaacctattaatttc
agacattacttcctcttttgagtggctccgtcaaggtatcctaccgttctaggaccatagccagacgctaaggctgagcaggttgtagttatgttggataattaaag

KanR

660 680 700 720 740

NsiI

ccctcgtcaaaaataaggttatcaagtgagaaatcaccatgagtgacgactgaatccggtgagaatggcaaaagtttatgcatttctttccagacttgttcaacagg
gggagcagtttttattccaatagttcactctttagtggtactcactgctgacttaggccactcttaccgttttcaaatacgtaaagaaaggtctgaacaagttgtcc

KanR

760 780 800 820 840

Bpu10I AjuI AsiSI PvuI

ccagccattacgctcgtcatcaaaatcactcgcatcaaccaaaccgttattcattcgtgattgcgcctgagcgagacgaaatacgcgatcgctgttaaaaggacaat
ggtcggtaatgcgagcagtagttttagtgagcgtagttggtttggcaataagtaagcactaacgcggactcgctctgctttatgcgctagcgacaattttcctgtta

KanR

860 880 900 920 940 960

XmaI SmaI TspMI BsmI SspI EcoNI BsmI

tacaaacaggaatcgaatgcaaccggcgcaggaacactgccagcgcatcaacaatattttcacctgaatcaggatattcttctaatacctggaatgctgttttcccg
atgtttgtccttagcttacgttggccgcgtccttgtgacggtcgcgtagttgttataaaagtggacttagtcctataagaagattatggaccttacgacaaaagggc

KanR

980 1,000 1,020 1,040 1,060

NsiI

gggatcgcagtggtgagtaaccatgcatcatcaggagtacggataaaatgcttgatggtcggaagaggcataaattccgtcagccagtttagtctgaccatctcatc
ccctagcgtcaccactcattggtacgtagtagtcctcatgcctattttacgaactaccagccttctccgtatttaaggcagtcggtcaaatcagactggtagagtag

KanR

1,080 1,100 1,120 1,140 1,160

BspDI ClaI

tgtaacatcattggcaacgctacctttgccatgtttcagaaacaactctggcgcatcgggcttcccatacaatcgatagattgtcgcacctgattgcccgacattat
acattgtagtaaccgttgcgatggaaacggtacaaagtctttgttgagaccgcgtagcccgaagggtatgttagctatctaacagcgtggactaacgggctgtaata

KanR

1,180 1,200 1,220 1,240 1,260 1,280

NruI

cgcgagcccatttatacccatataaatcagcatccatgttggaatttaatcgcggcctagagcaagacgtttcccgttgaatatggctcataacaccccttgtatta
gcgctcgggtaaatatgggtatatttagtcgtaggtacaaccttaaattagcgccggatctcgttctgcaaagggcaacttataccgagtattgtggggaacataat

KanR

1,300 1,320 1,340 1,360 1,380

ctgtttatgtaagcagacagttttattgttcatgaccaaaatcccttaacgtgagttttcgttccactgagcgtcagaccccgtagaaaagatcaaaggatcttctt
gacaaatacattcgtctgtcaaaataacaagtactggttttagggaattgcactcaaaagcaaggtgactcgcagtctggggcatcttttctagtttcctagaagaa

1,400 1,420 1,440 1,460 1,480

AcuI

gagatcctttttttctgcgcgtaatctgctgcttgcaaacaaaaaaaccaccgctaccagcggtggtttgtttgccggatcaagagctaccaactctttttccgaag
ctctaggaaaaaaagacgcgcattagacgacgaacgtttgtttttttggtggcgatggtcgccaccaaacaaacggcctagttctcgatggttgagaaaaaggcttc

ori

1,500 1,520 1,540 1,560 1,580 1,600

gtaactggcttcagcagagcgcagataccaaatactgtccttctagtgtagccgtagttaggccaccacttcaagaactctgtagcaccgcctacatacctcgctct
cattgaccgaagtcgtctcgcgtctatggtttatgacaggaagatcacatcggcatcaatccggtggtgaagttcttgagacatcgtggcggatgtatggagcgaga

ori

1,620 1,640 1,660 1,680 1,700

AlwNI

gctaatcctgttaccagtggctgctgccagtggcgataagtcgtgtcttaccgggttggactcaagacgatagttaccggataaggcgcagcggtcgggctgaacgg
cgattaggacaatggtcaccgacgacggtcaccgctattcagcacagaatggcccaacctgagttctgctatcaatggcctattccgcgtcgccagcccgacttgcc

ori

1,720 1,740 1,760 1,780 1,800

ggggttcgtgcacacagcccagcttggagcgaacgacctacaccgaactgagatacctacagcgtgagctatgagaaagcgccacgcttcccgaagggagaaaggcg
ccccaagcacgtgtgtcgggtcgaacctcgcttgctggatgtggcttgactctatggatgtcgcactcgatactctttcgcggtgcgaagggcttccctctttccgc

ori

1,820 1,840 1,860 1,880 1,900 1,920

BssSαI
BssSI

gacaggtatccggtaagcggcagggtcggaacaggagagcgcacgagggagcttccagggggaaacgcctggtatctttatagtcctgtcgggtttcgccacctctg
ctgtccataggccattcgccgtcccagccttgtcctctcgcgtgctccctcgaaggtccccctttgcggaccatagaaatatcaggacagcccaaagcggtggagac

ori

1,940 1,960 1,980 2,000 2,020

acttgagcgtcgatttttgtgatgctcgtcaggggggcggagcctatggaaaaacgccagcaacgcggcctttttacggttcctggccttttgctggccttttgctc
tgaactcgcagctaaaaacactacgagcagtccccccgcctcggatacctttttgcggtcgttgcgccggaaaaatgccaaggaccggaaaacgaccggaaaacgag

ori

2,040 2,060 2,080 2,100 2,120 2,140

PciI

acatgttctttcctgcgttatcccctgattctgtggataaccgtattaccgcctttgagtgagctgataccgctcgccgcagccgaacgaccgagcgcagcgagtca
tgtacaagaaaggacgcaataggggactaagacacctattggcataatggcggaaactcactcgactatggcgagcggcgtcggcttgctggctcgcgtcgctcagt

2,160 2,180 2,200 2,220 2,240

BspQI
SapI

gtgagcgaggaagcggaagagcgcctgatgcggtattttctccttacgcatctgtgcggtatttcacaccgcatatatggtgcactctcagtacaatctgctctgat
cactcgctccttcgccttctcgcggactacgccataaaagaggaatgcgtagacacgccataaagtgtggcgtatataccacgtgagagtcatgttagacgagacta

bom

2,260 2,280 2,300 2,320 2,340

Tth111I
PflFI
BstZ17I BsaAI

gccgcatagttaagccagtatacactccgctatcgctacgtgactgggtcatggctgcgccccgacacccgccaacacccgctgacgcgccctgacgggcttgtctg
cggcgtatcaattcggtcatatgtgaggcgatagcgatgcactgacccagtaccgacgcggggctgtgggcggttgtgggcgactgcgcgggactgcccgaacagac

bom

2,360 2,380 2,400 2,420 2,440 2,460

PfoI BcgI

ctcccggcatccgcttacagacaagctgtgaccgtctccgggagctgcatgtgtcagaggttttcaccgtcatcaccgaaacgcgcgaggcagctgcggtaaagctc
gagggccgtaggcgaatgtctgttcgacactggcagaggccctcgacgtacacagtctccaaaagtggcagtagtggctttgcgcgctccgtcgacgccatttcgag

rop

2,480 2,500 2,520 2,540 2,560

XmnI

atcagcgtggtcgtgaagcgattcacagatgtctgcctgttcatccgcgtccagctcgttgagtttctccagaagcgttaatgtctggcttctgataaagcgggcca
tagtcgcaccagcacttcgctaagtgtctacagacggacaagtaggcgcaggtcgagcaactcaaagaggtcttcgcaattacagaccgaagactatttcgcccggt

rop

2,580 2,600 2,620 2,640 2,660

tgttaagggcggtttttcctgtttggtcactgatgcctccgtgtaagggggatttctgttcatgggggtaatgataccgatgaaacgagagaggatgctcacgata
acaattcccgccaaaaaggacaaaccagtgactacggaggcacattccccctaaagacaagtacccccattactatggctactttgctctctcctacgagtgctat

rop

2,680 2,700 2,720 2,740 2,760 2,780

cgggttactgatgatgaacatgcccggttactggaacgttgtgagggtaaacaactggcggtatggatgcggcgggaccagagaaaaatcactcagggtcaatgcca
gcccaatgactactacttgtacgggccaatgaccttgcaacactcccatttgttgaccgccatacctacgccgccctggtctctttttagtgagtcccagttacggt

2,800 2,820 2,840 2,860 2,880

AfeI
gcgcttcgttaatacagatgtaggtgttccacagggtagccagcagcatcctgcgatgcagatccggaacataatggtgcagggcgctgacttccgcgtttccagac
cgcgaagcaattatgtctacatccacaaggtgtcccatcggtcgtcgtaggacgctacgtctaggccttgtattaccacgtcccgcgactgaaggcgcaaaggtctg

2,900 2,920 2,940 2,960 2,980

Bpu10I
tttacgaaacacggaaaccgaagaccattcatgttgttgctcaggtcgcagacgttttgcagcagcagtcgcttcacgttcgctcgcgtatcggtgattcattctgc
aaatgctttgtgcctttggcttctggtaagtacaacaacgagtccagcgtctgcaaaacgtcgtcgtcagcgaagtgcaagcgagcgcatagccactaagtaagacg

3,000 3,020 3,040 3,060 3,080 3,100

PpuMI
taaccagtaaggcaaccccgccagcctagccgggtcctcaacgacaggagcacgatcatgctagtcatgccccgcgcccaccggaaggagctgactgggttgaaggc
attggtcattccgttggggcggtcggatcggcccaggagttgctgtcctcgtgctagtacgatcagtacggggcgcgggtggccttcctcgactgacccaacttccg

3,120 3,140 3,160 3,180 3,200

tctcaagggcatcggtcgagatcccggtgcctaatgagtgagctaacttacattaattgcgttgcgctcactgcccgctttccagtcgggaaacctgtcgtgccagc
agagttcccgtagccagctctagggccacggattactcactcgattgaatgtaattaacgcaacgcgagtgacgggcgaaaggtcagccctttggacagcacggtcg
lacI

3,220 3,240 3,260 3,280 3,300

BsaXI
tgcattaatgaatcggccaacgcgcggggagaggcggtttgcgtattgggcgccagggtggtttttcttttcaccagtgagacgggcaacagctgattgcccttcac
acgtaattacttagccggttgcgcgcccctctccgccaaacgcataacccgcggtcccaccaaaaagaaaagtggtcactctgcccgttgtcgactaacgggaagtg
lacI

3,320 3,340 3,360 3,380 3,400 3,420

HincII
HpaI
cgcctggccctgagagagttgcagcaagcggtccacgctggtttgccccagcaggcgaaaatcctgtttgatggtggttaacggcgggatataacatgagctgtctt
gcggaccgggactctctcaacgtcgttcgccaggtgcgaccaaacggggtcgtccgcttttaggacaaactaccaccaattgccgccctatattgtactcgacagaa
lacI

3,440 3,460 3,480 3,500 3,520

BsrDI
BssHII
cggtatcgtcgtatcccactaccgagatgtccgcaccaacgcgcagcccggactcggtaatggcgcgcattgcgcccagcgccatctgatcgttggcaaccagcatc
gccatagcagcatagggtgatggctctacaggcgtggttgcgcgtcgggcctgagccattaccgcgcgtaacgcgggtcgcggtagactagcaaccgttggtcgtag
lacI

3,540 3,560 3,580 3,600 3,620

BcgI

gcagtgggaacgatgccctcattcagcatttgcatggtttgttgaaaaccggacatggcactccagtcgccttcccgttccgctatcggctgaatttgattgcgagt
cgtcacccttgctacgggagtaagtcgtaaacgtaccaaacaacttttggcctgtaccgtgaggtcagcggaagggcaaggcgatagccgacttaaactaacgctca

lacI

3,640 3,660 3,680 3,700 3,720 3,740

ApaI
PspOMI NmeAIII BstEII

gagatatttatgccagccagccagacgcagacgcgccgagacagaacttaatgggcccgctaacagcgcgatttgctggtgacccaatgcgaccagatgctccacgc
ctctataaatacggtcggtcggtctgcgtctgcgcggctctgtcttgaattacccgggcgattgtcgcgctaaacgaccactgggttacgctggtctacgaggtgcg

lacI

3,760 3,780 3,800 3,820 3,840

ccagtcgcgtaccgtcttcatgggagaaaataatactgttgatgggtgtctggtcagagacatcaagaaataacgccggaacattagtgcaggcagcttccacagca
ggtcagcgcatggcagaagtaccctcttttattatgacaactacccacagaccagtctctgtagttctttattgcggccttgtaatcacgtccgtcgaaggtgtcgt

lacI

3,860 3,880 3,900 3,920 3,940

BsrDI BclI MluI

atggcatcctggtcatccagcggatagttaatgatcagcccactgacgcgttgcgcgagaagattgtgcaccgccgctttacaggcttcgacgccgcttcgttctac
taccgtaggaccagtaggtcgcctatcaattactagtcgggtgactgcgcaacgcgctcttctaacacgtggcggcgaaatgtccgaagctgcggcgaagcaagatg

lacI

3,960 3,980 4,000 4,020 4,040 4,060

catcgacaccaccacgctggcacccagttgatcggcgcgagatttaatcgccgcgacaatttgcgacggcgcgtgcagggccagactggaggtggcaacgccaatca
gtagctgtggtggtgcgaccgtgggtcaactagccgcgctctaaattagcggcgctgttaaacgctgccgcgcacgtcccggtctgacctccaccgttgcggttagt

lacI

4,080 4,100 4,120 4,140 4,160

gcaacgactgtttgcccgccagttgttgtgccacgcggttgggaatgtaattcagctccgccatcgccgcttccactttttcccgcgttttcgcagaaacgtggctg
cgttgctgacaaacgggcggtcaacaacacggtgcgccaacccttacattaagtcgaggcggtagcggcgaaggtgaaaaagggcgcaaaagcgtctttgcaccgac

lacI

4,180 4,200 4,220 4,240 4,260 4,280

BstAPI

gcctggttcaccacgcgggaaacggtctgataagagacaccggcatactctgcgacatcgtataacgttactggtttcacattcaccaccctgaattgactctcttc
cggaccaagtggtgcgccctttgccagactattctctgtggccgtatgagacgctgtagcatattgcaatgaccaaagtgtaagtggtgggacttaactgagagaag

lacI lacI promoter

4,300 4,320 4,340 4,360 4,380

PflMI PfoI EcoNI

cgggcgctatcatgccataccgcgaaaggttttgcgccattcgatggtgtccgggatctcgacgctctcccttatgcgactcctgcattaggaagcagcccagtagt
gcccgcgatagtacggtatggcgctttccaaaacgcggtaagctaccacaggccctagagctgcgagagggaatacgctgaggacgtaatccttcgtcgggtcatca

lacI promoter

4,400 4,420 4,440 4,460 4,480

SphI

aggttgaggccgttgagcaccgccgccgcaaggaatggtgcatgcaaggagatggcgcccaacagtcccccggccacggggcctgccaccatacccacgccgaaaca
tccaactccggcaactcgtggcggcggcgttccttaccacgtacgttcctctaccgcgggttgtcagggggccggtgccccggacggtggtatgggtgcggctttgt

4,500 4,520 4,540 4,560 4,580 4,600

NaeI
AfeI SgrAI NgoMIV

agcgctcatgagcccgaagtggcgagcccgatcttccccatcggtgatgtcggcgatataggcgccagcaaccgcacctgtggcgccggtgatgccggccacgatgc
tcgcgagtactcgggcttcaccgctcgggctagaaggggtagccactacagccgctatatccgcggtcgttggcgtggacaccgcggccactacggccggtgctacg

4,620 4,640 4,660 4,680 4,700

BspDI
ClaI XbaI

gtccggcgtagaggatcgagatcgatctcgatcccgcgaaattaatacgactcactataggggaattgtgagcggataacaattcccctctagaaataattttgttt
caggccgcatctcctagctctagctagagctagggcgctttaattatgctgagtgatatccccttaacactcgcctattgttaaggggagatctttattaaaacaaa

T7 promoter lac operator

4,720 4,740 4,760 4,780 4,800

BarI

aactttaagaaggagatatacataATGAGTAAAGGAGAAGAACTTTTCACTGGAGTTGTCCCAATTCTTGTTGAATTAGATGGTGATGTTAATGGGCACAAATTTTC
ttgaaattcttcctctatatgtatTACTCATTTCCTCTTCTTGAAAAGTGACCTCAACAGGGTTAAGAACAACTTAATCTACCACTACAATTACCCGTGTTTAAAAG

RBS superfolder GFP

4,820 4,840 4,860 4,880 4,900 4,920

MscI
NcoI

TGTCCGTGGAGAGGGTGAAGGTGATGCAACAAACGGAAAACTTACCCTTAAATTTATTTGCACTACTGGAAAACTACCTGTTCCATGGCCAACACTTGTCACTACTT
ACAGGCACCTCTCCCACTTCCACTACGTTGTTTGCCTTTTGAATGGGAATTTAAATAAACGTGATGACCTTTTGATGGACAAGGTACCGGTTGTGAACAGTGATGAA

superfolder GFP

4,940 4,960 4,980 5,000 5,020

BarI BsrGI

TAACTTATGGTGTTCAATGCTTTTCCCGTTATCCGGATCACATGAAACGGCATGACTTTTTCAAGAGTGCCATGCCCGAAGGTTATGTACAGGAACGCACTATATCT
ATTGAATACCACAAGTTACGAAAAGGGCAATAGGCCTAGTGTACTTTGCCGTACTGAAAAAGTTCTCACGGTACGGGCTTCCAATACATGTCCTTGCGTGATATAGA

superfolder GFP

5,040 5,060 5,080 5,100 5,120

PpuMI MluI AcuI BaeI DraI

TTCAAAGATGACGGGACCTACAAGACGCGTGCTGAAGTCAAGTTTGAAGGTGATACCCTTGTTAATCGTATCGAGTTAAAAGGTATTGATTTTAAAGAAGATGGAAA
AAGTTTCTACTGCCCTGGATGTTCTGCGCACGACTTCAGTTCAAACTTCCACTATGGGAACAATTAGCATAGCTCAATTTTCCATAACTAAAATTTCTTCTACCTTT

superfolder GFP

5,140 5,160 5,180 5,200 5,220 5,240

PaeR7I PspXI XhoI TliI BstZ17I

CATTCTCGGACACAAACTCGAGTACAACTTTAACTCACACAATGTATACATCACGGCAGACAAACAAAAGAATGGAATCAAAGCTAACTTCAAAATTCGCCACAACG
GTAAGAGCCTGTGTTTGAGCTCATGTTGAAATTGAGTGTGTTACATATGTAGTGCCGTCTGTTTGTTTTCTTACCTTAGTTTCGATTGAAGTTTTAAGCGGTGTTGC

superfolder GFP

5,260 5,280 5,300 5,320 5,340

BamHI MfeI SalI HincII

TTGAAGATGGATCCGTTCAACTAGCAGACCATTATCAACAAAATACTCCAATTGGCGATGGCCCTGTCCTTTTACCAGACAACCATTACCTGTCGACACAATCTGTC
AACTTCTACCTAGGCAAGTTGATCGTCTGGTAATAGTTGTTTTATGAGGTTAACCGCTACCGGGACAGGAAAATGGTCTGTTGGTAATGGACAGCTGTGTTAGACAG

superfolder GFP

5,360 5,380 5,400 5,420 5,440

BstBI Eco53kI SacI PaeR7I PspXI XhoI TliI

CTTTCGAAAGATCCCAACGAAAAGCGTGACCACATGGTCCTTCTTGAGTTTGTAACTGCTGCTGGGATTACACATGGCATGGATGAGCTCTACAAATGActcgagca
GAAAGCTTTCTAGGGTTGCTTTTCGCACTGGTGTACCAGGAAGAACTCAAACATTGACGACGACCCTAATGTGTACCGTACCTACTCGAGATGTTTACTgagctcgt

superfolder GFP

5,460 5,480 5,500 5,520 5,540 5,560

AvrII BlpI

ccaccaccaccaccaccaccactaattgattaatacctaggctgctaaacaaagcccgaaaggaagctgagttggctgctgccaccgctgagcaataactagcataa
ggtggtggtggtggtggtggtgattaactaattatggatccgacgatttgtttcgggctttccttcgactcaaccgacgacggtggcgactcgttattgatcgtatt

8xHis

5,580 5,600 5,620 5,640 5,660

ccccttggggcctctaaacgggtcttgaggggttttttgctgaaaggaggaactatatccggat
ggggaaccccggagatttgcccagaactccccaaaaaacgactttcctccttgatataggccta

T7 terminator

5,680 5,690 5,700 5,710 5,720 5,730

pT7_rplA (3067 bp)

AatII
ZraI
BsaHI

GACGAAAGGGCCTCGTGATACGCCTATTTTTATAGGTTAATGTCATGATAATAATGGTTTCTTAGACGTCAGGTGGCACTTTTCGGGGAAATGTGCGCGGAACCCCT
CTGCTTTCCCGGAGCACTATGCGGATAAAAATATCCAATTACAGTACTATTATTACCAAAGAATCTGCAGTCCACCGTGAAAAGCCCCTTTACACGCGCCTTGGGGA

20 40 60 80 100

BciVI
BsrBI SspI EarI

ATTTGTTTATTTTTCTAAATACATTCAAATATGTATCCGCTCATGAGACAATAACCCTGATAAATGCTTCAATAATATTGAAAAAGGAAGAGTATGAGTATTCAACA
TAAACAAATAAAAAGATTTATGTAAGTTTATACATAGGCGAGTACTCTGTTATTGGGACTATTTACGAAGTTATTATAACTTTTTCCTTCTCATACTCATAAGTTGT

120 140 160 180 200

BaeGI
ApaLI

TTTCCGTGTCGCCCTTATTCCCTTTTTTGCGGCATTTTGCCTTCCTGTTTTTGCTCACCCAGAAACGCTGGTGAAAGTAAAAGATGCTGAAGATCAGTTGGGTGCAC
AAAGGCACAGCGGGAATAAGGGAAAAAACGCCGTAAAACGGAAGGACAAAAACGAGTGGGTCTTTGCGACCACTTTCATTTTCTACGACTTCTAGTCAACCCACGTG

220 240 260 280 300 320

XmnI

GAGTGGGTTACATCGAACTGGATCTCAACAGCGGTAAGATCCTTGAGAGTTTTCGCCCCGAAGAACGTTTTCCAATGATGAGCACTTTTAAAGTTCTGCTATGTGGC
CTCACCCAATGTAGCTTGACCTAGAGTTGTCGCCATTCTAGGAACTCTCAAAAGCGGGGCTTCTTGCAAAAGGTTACTACTCGTGAAAATTTCAAGACGATACACCG

AmpR

340 360 380 400 420

BsaHI BcgI ScaI

GCGGTATTATCCCGTATTGACGCCGGGCAAGAGCAACTCGGTCGCCGCATACACTATTCTCAGAATGACTTGGTTGAGTACTCACCAGTCACAGAAAAGCATCTTAC
CGCCATAATAGGGCATAACTGCGGCCCGTTCTCGTTGAGCCAGCGGCGTATGTGATAAGAGTCTTACTGAACCAACTCATGAGTGGTCAGTGTCTTTTCGTAGAATG

AmpR

440 460 480 500 520

PvuI

GGATGGCATGACAGTAAGAGAATTATGCAGTGCTGCCATAACCATGAGTGATAACACTGCGGCCAACTTACTTCTGACAACGATCGGAGGACCGAAGGAGCTAACCG
CCTACCGTACTGTCATTCTCTTAATACGTCACGACGGTATTGGTACTCACTATTGTGACGCCGGTTGAATGAAGACTGTTGCTAGCCTCCTGGCTTCCTCGATTGGC

AmpR

540 560 580 600 620 640

CTTTTTTGCACAACATGGGGGATCATGTAACTCGCCTTGATCGTTGGGAACCGGAGCTGAATGAAGCCATACCAAACGACGAGCGTGACACCACGATGCCTGTAGCA
GAAAAAACGTGTTGTACCCCCTAGTACATTGAGCGGAACTAGCAACCCTTGGCCTCGACTTACTTCGGTATGGTTTGCTGCTCGCACTGTGGTGCTACGGACATCGT

AmpR

660 680 700 720 740

FspI NmeAIII

ATGGCAACAACGTTGCGCAAACTATTAACTGGCGAACTACTTACTCTAGCTTCCCGGCAACAATTAATAGACTGGATGGAGGCGGATAAAGTTGCAGGACCACTTCT
TACCGTTGTTGCAACGCGTTTGATAATTGACCGCTTGATGAATGAGATCGAAGGGCCGTTGTTAATTATCTGACCTACCTCCGCCTATTTCAACGTCCTGGTGAAGA

AmpR

760 780 800 820 840

BsaI
BglI BsrFI BpmI BmrI

GCGCTCGGCCCTTCCGGCTGGCTGGTTTATTGCTGATAAATCTGGAGCCGGTGAGCGTGGGTCTCGCGGTATCATTGCAGCACTGGGGCCAGATGGTAAGCCCTCCC
CGCGAGCCGGGAAGGCCGACCGACCAAATAACGACTATTTAGACCTCGGCCACTCGCACCCAGAGCGCCATAGTAACGTCGTGACCCCGGTCTACCATTCGGGAGGG

AmpR

860 880 900 920 940 960

AhdI BanI

GTATCGTAGTTATCTACACGACGGGGAGTCAGGCAACTATGGATGAACGAAATAGACAGATCGCTGAGATAGGTGCCTCACTGATTAAGCATTGGTAACTGTCAGAC
CATAGCATCAATAGATGTGCTGCCCCTCAGTCCGTTGATACCTACTTGCTTTATCTGTCTAGCGACTCTATCCACGGAGTGACTAATTCGTAACCATTGACAGTCTG

AmpR

980 1,000 1,020 1,040 1,060

CAAGTTTACTCATATATACTTTAGATTGATTTAAAACTTCATTTTTAATTTAAAAGGATCTAGGTGAAGATCCTTTTTGATAATCTCATGACCAAAATCCCTTAACG
GTTCAAATGAGTATATATGAAATCTAACTAAATTTTGAAGTAAAAATTAAATTTTCCTAGATCCACTTCTAGGAAAAACTATTAGAGTACTGGTTTTAGGGAATTGC

ColE1 origin

1,080 1,100 1,120 1,140 1,160

TGAGTTTTCGTTCCACTGAGCGTCAGACCCCGTAGAAAAGATCAAAGGATCTTCTTGAGATCCTTTTTTTCTGCGCGTAATCTGCTGCTTGCAAACAAAAAAACCAC
ACTCAAAAGCAAGGTGACTCGCAGTCTGGGGCATCTTTTCTAGTTTCCTAGAAGAACTCTAGGAAAAAAAGACGCGCATTAGACGACGAACGTTTGTTTTTTTGGTG

ColE1 origin

1,180 1,200 1,220 1,240 1,260 1,280

CGCTACCAGCGGTGGTTTGTTTGCCGGATCAAGAGCTACCAACTCTTTTTCCGAAGGTAACTGGCTTCAGCAGAGCGCAGATACCAAATACTGTTCTTCTAGTGTAG
GCGATGGTCGCCACCAAACAAACGGCCTAGTTCTCGATGGTTGAGAAAAAGGCTTCCATTGACCGAAGTCGTCTCGCGTCTATGGTTTATGACAAGAAGATCACATC

ColE1 origin

1,300 1,320 1,340 1,360 1,380

AlwNI MmeI

CCGTAGTTAGGCCACCACTTCAAGAACTCTGTAGCACCGCCTACATACCTCGCTCTGCTAATCCTGTTACCAGTGGCTGCTGCCAGTGGCGATAAGTCGTGTCTTAC
GGCATCAATCCGGTGGTGAAGTTCTTGAGACATCGTGGCGGATGTATGGAGCGAGACGATTAGGACAATGGTCACCGACGACGGTCACCGCTATTCAGCACAGAATG

ColE1 origin

1,400 1,420 1,440 1,460 1,480

BaeGI
ApaLI BseYI

CGGGTTGGACTCAAGACGATAGTTACCGGATAAGGCGCAGCGGTCGGGCTGAACGGGGGGTTCGTGCACACAGCCCAGCTTGGAGCGAACGACCTACACCGAACTGA
GCCCAACCTGAGTTCTGCTATCAATGGCCTATTCCGCGTCGCCAGCCCGACTTGCCCCCCAAGCACGTGTGTCGGGTCGAACCTCGCTTGCTGGATGTGGCTTGACT

ColE1 origin

1,500 1,520 1,540 1,560 1,580 1,600

MmeI BciVI

GATACCTACAGCGTGAGCTATGAGAAAGCGCCACGCTTCCCGAAGGGAGAAAGGCGGACAGGTATCCGGTAAGCGGCAGGGTCGGAACAGGAGAGCGCACGAGGGAG
CTATGGATGTCGCACTCGATACTCTTTCGCGGTGCGAAGGGCTTCCCTCTTTCCGCCTGTCCATAGGCCATTCGCCGTCCCAGCCTTGTCCTCTCGCGTGCTCCCTC

ColE1 origin

1,620 1,640 1,660 1,680 1,700

DrdI

CTTCCAGGGGGAAACGCCTGGTATCTTTATAGTCCTGTCGGGTTTCGCCACCTCTGACTTGAGCGTCGATTTTTGTGATGCTCGTCAGGGGGGCGGAGCCTATGGAA
GAAGGTCCCCCTTTGCGGACCATAGAAATATCAGGACAGCCCAAAGCGGTGGAGACTGAACTCGCAGCTAAAAACACTACGAGCAGTCCCCCCGCCTCGGATACCTT

ColE1 origin

1,720 1,740 1,760 1,780 1,800

AflIII
PciI TfiI

AAACGCCAGCAACGCGGCCTTTTTACGGTTCCTGGCCTTTTGCTGGCCTTTTGCTCACATGTTCTTTCCTGCGTTATCCCCTGATTCTGTGGATAACCGTATTACCG
TTTGCGGTCGTTGCGCCGGAAAAATGCCAAGGACCGGAAAACGACCGGAAAACGAGTGTACAAGAAAGGACGCAATAGGGGACTAAGACACCTATTGGCATAATGGC

ColE1 origin

1,820 1,840 1,860 1,880 1,900 1,920

BspQI
SapI
BsrBI EarI BsaXI

CCTTTGAGTGAGCTGATACCGCTCGCCGCAGCCGAACGACCGAGCGCAGCGAGTCAGTGAGCGAGGAAGCGGAAGAGCGCCCAATACGCAAACCGCCTCTCCCCGCG
GGAAACTCACTCGACTATGGCGAGCGGCGTCGGCTTGCTGGCTCGCGTCGCTCAGTCACTCGCTCCTTCGCCTTCTCGCGGGTTATGCGTTTGGCGGAGAGGGGCGC

1,940 1,960 1,980 2,000 2,020

TfiI PvuII XbaI

CGTTGGCCGATTCATTAATGCAGCTGGCACGACAGGTTTCCCGACTGGAAAGCGGGCAGTGAGCGCAACGCAATtaatacgactcactataggcccctctagaaata
GCAACCGGCTAAGTAATTACGTCGACCGTGCTGTCCAAAGGGCTGACCTTTCGCCCGTCACTCGCGTTGCGTTAattatgctgagtgatatccggggagatctttat

T7P

2,040 2,060 2,080 2,100 2,120 2,140

SphI

atttttgtttaacttcataggaggccgcaaatggctaaactgaccaagcgcatgcgtgttatccgcgagaaagttgatgcaaccaaacagtacgacatcaacgaagct
taaaacaaattgaagtatcctccggcgtttaccgatttgactggttcgcgtacgcacaataggcgctctttcaactacgttggtttgtcatgctgtagttgcttcga

rplA

2,160 2,180 2,200 2,220 2,240

BsaAI
HincII XcmI
ApoI NmeAIII HpaI BsiWI

atcgcactgctgaaagagctggcgactgctaaattcgtagaaagcgtggacgtagctgttaacctcggcatcgacgctcgtaaatctgaccagaacgtacgtggtgc
tagcgtgacgactttctcgaccgctgacgatttaagcatctttcgcacctgcatcgacaattggagccgtagctgcgagcatttagactggtcttgcatgcaccacg

rplA

2,260 2,280 2,300 2,320 2,340

PsrI PstI

aactgtactgccgcacggtactggccgttccgttcgcgtagccgtatttacccaaggtgcaaacgctgaagctgctaaagctgcaggcgcagaactggtaggtatgg
ttgacatgacggcgtgccatgaccggcaaggcaagcgcatcggcataaatgggttccacgtttgcgacttcgacgatttcgacgtccgcgtcttgaccatccatacc

rplA

2,360 2,380 2,400 2,420 2,440 2,460

SfiI
PvuII
BseYI PflMI
BglII BspEI MscI BglI AlwNI

aagatctggctgaccagatcaagaaaggcgaaatgaactttgacgttgttattgcttctccggatgcaatgcgcgttgttggccagctgggccaggttctgggtccg
ttctagaccgactggtctagttctttccgctttacttgaaactgcaacaataacgaagaggcctacgttacgcgcaacaaccggtcgacccggtccaagacccaggc

rplA

2,480 2,500 2,520 2,540 2,560

MscI

cgcggcctgatgccaaacccgaaagtgggtactgtaacaccgaacgttgctgaagcggttaaaaacgctaaagctggccaggttcgttaccgtaacgacaaaaacgg
gcgccggactacggtttgggctttcacccatgacattgtggcttgcaacgacttcgccaatttttgcgatttcgaccggtccaagcaatggcattgctgtttttgcc

rplA

2,580 2,600 2,620 2,640 2,660

XcmI

catcatccacaccaccatcggtaaagtggactttgacgctgacaaactgaaagaaaacctggaagctctgctggttgcgctgaaaaaagcaaaaccgactcaggcga
gtagtaggtgtggtggtagccatttcacctgaaactgcgactgtttgactttcttttggaccttcgagacgaccaacgcgactttttttcgttttggctgagtccgct

rplA

2,680 2,700 2,720 2,740 2,760 2,780

BstXI
NcoI
BtgI
BspMI
BfuAI
BsrGI AarI BstAPI HincII BsgI Bpu10I AfeI

aaggcgtgtacatcaagaaagttagcatctccaccaccatgggtgcaggtgttgcagttgaccaggctggcctgagcgcttctgtaaactaactagcataaccccttttccgcacatgtagttctttcaatcgtagaggtggtggtacccacgtccacaacgtcaactggtccgaccggactcgcgaagacatttgattgatcgtattggggaa

rplA

2,800 2,820 2,840 2,860 2,880

DrdI

ggggcctctaaacgggtcttgaggggttttttgTTAAGCCAGCCCCGACACCCGCCAACACCCGCTGACGCGCCCTGACGGGCTTGTCTGCTCCCGGCATCCGCTTA
ccccggagatttgcccagaactccccaaaaaacAATTCGGTCGGGGCTGTGGGCGGTTGTGGGCGACTGCGCGGGACTGCCCGAACAGACGAGGGCCGTAGGCGAAT

2,900 2,920 2,940 2,960 2,980

BsmBI
PfoI BsmBI

CAGACAAGCTGTGACCGTCTCCGGGAGCTGCATGTGTCAGAGGTTTTCACCGTCATCACCGAAACGCGCGA
GTCTGTTCGACACTGGCAGAGGCCCTCGACGTACACAGTCTCCAAAAGTGGCAGTAGTGGCTTTGCGCGCT

3,000 3,010 3,020 3,030 3,040 3,050 3,060

pUC19_T7_LacZ (5539 bp)

```
BsaXI
gcgcccaatacgcaaaccgcctctccccgcgcgttggccgattcattaatgcagctggcacgacaggtttcccgactggaaagcgggcagtgagcgcaacgcaataa
cgcgggttatgcgtttggcggagaggggcgcgcaaccggctaagtaattacgtcgaccgtgctgtccaaagggctgacctttcgcccgtcactcgcgttgcgttatt
20 40 60 80 100
```

```
PluTI SgrAI SfoI NarI KasI PluTI SfoI NarI KasI AfeI NaeI NgoMIV
gcgctcatgagcccgaagtggcgagcccgatcttccccatcggtgatgtcggcgatataggcgccagcaaccgcacctgtggcgccggtgatgccggccacgatgcg
cgcgagtactcgggcttcaccgctcgggctagaaggggtagccactacagccgctatatccgcggtcgttggcgtggacaccgcggccactacggccggtgctacgc
120 140 160 180 200
```

```
BspDI ClaI Eco0109I XbaI
tccggcgtagaggatcgagatcgatctcgatcccgcgaaattaatacgactcactataggcccctctagaaataattttgtttaacttcataggaggccgcaaATGA
aggccgcatctcctagctctagctagagctagggcgctttaattatgctgagtgatatccggggagatctttattaaaacaaattgaagtatcctccggcgttTACT
T7 promoter
220 240 260 280 300 320
```

```
CCATGATTACGGATTCACTGGCCGTCGTTTTACAACGTCGTGACTGGGAAAACCCTGGCGTTACCCAACTTAATCGCCTTGCAGCACATCCCCCTTTCGCCAGCTGG
GGTACTAATGCCTAAGTGACCGGCAGCAAAATGTTGCAGCACTGACCCTTTTGGGACCGCAATGGGTTGAATTAGCGGAACGTCGTGTAGGGGGAAAGCGGTCGACC
LacZ
340 360 380 400 420
```

```
FspI
CGTAATAGCGAAGAGGCCCGCACCGATCGCCCTTCCCAACAGTTGCGCAGCCTGAATGGCGAATGGCGCTTTGCCTGGTTTCCGGCACCAGAAGCGGTGCCGGAAAG
GCATTATCGCTTCTCCGGGCGTGGCTAGCGGGAAGGGTTGTCAACGCGTCGGACTTACCGCTTACCGCGAAACGGACCAAAGGCCGTGGTCTTCGCCACGGCCTTTC
LacZ alpha
LacZ
440 460 480 500 520
```

```
Bsu36I BaeI
CTGGCTGGAGTGCGATCTTCCTGAGGCCGATACTGTCGTCGTCCCCTCAAACTGGCAGATGCACGGTTACGATGCGCCCATCTACACCAACGTgACCTATCCCATTA
GACCGACCTCACGCTAGAAGGACTCCGGCTATGACAGCAGCAGGGGAGTTTGACCGTCTACGTGCCAATGCTACGCGGGTAGATGTGGTTGCACTGGATAGGGTAAT
LacZ
540 560 580 600 620 640
```

NmeAIII

CGGTCAATCCGCCGTTTGTTCCCACGGAGAATCCGACGGGTTGTTACTCGCTCACATTTAATGTTGATGAAAGCTGGCTACAGGAAGGCCAGACGCGAATTATTTTT
GCCAGTTAGGCGGCAAACAAGGGTGCCTCTTAGGCTGCCCAACAATGAGCGAGTGTAAATTACAACTACTTTCGACCGATGTCCTTCCGGTCTGCGCTTAATAAAAA

LacZ

660 680 700 720 740

HpaI ApoI Bpu10I

GATGGCGTTAACTCGGCGTTTCATCTGTGGTGCAACGGGCGCTGGGTCGGTTACGGCCAGGACAGTCGTTTGCCGTCTGAATTTGACCTGAGCGCATTTTTACGCGC
CTACCGCAATTGAGCCGCAAAGTAGACACCACGTTGCCCGCGACCCAGCCAATGCCGGTCCTGTCAGCAAACGGCAGACTTAAACTGGACTCGCGTAAAAATGCGCG

LacZ

760 780 800 820 840

AatII
ZraI

CGGAGAAAACCGCCTCGCGGTGATGGTGCTGCGcTGGAGTGACGGCAGTTATCTGGAAGATCAGGATATGTGGCGGATGAGCGGCATTTTCCGTGACGTCTCGTTGC
GCCTCTTTTGGCGGAGCGCCACTACCACGACGCgACCTCACTGCCGTCAATAGACCTTCTAGTCCTATACACCGCCTACTCGCCGTAAAAGGCACTGCAGAGCAACG

LacZ

860 880 900 920 940 960

TatI

TGCATAAACCGACTACACAAATCAGCGATTTCCATGTTGCCACTCGCTTTAATGATGATTTCAGCCGCGCTGTACTGGAGGCTGAAGTTCAGATGTGCGGCGAGTTG
ACGTATTTGGCTGATGTGTTTAGTCGCTAAAGGTACAACGGTGAGCGAAATTACTACTAAAGTCGGCGCGACATGACCTCCGACTTCAAGTCTACACGCCGCTCAAC

LacZ

980 1,000 1,020 1,040 1,060

BspMI BspDI
BfuAI ClaI

CGTGACTACCTACGGGTAACAGTTTCTTTATGGCAGGGTGAAACGCAGGTCGCCAGCGGCACCGCGCCTTTCGGCGGTGAAATTATCGATGAGCGTGGTGGTTATGC
GCACTGATGGATGCCCATTGTCAAAGAAATACCGTCCCACTTTGCGTCCAGCGGTCGCCGTGGCGCGGAAAGCCGCCACTTTAATAGCTACTCGCACCACCAATACG

LacZ

1,080 1,100 1,120 1,140 1,160

CGATCGCGTCACACTACGTCTGAACGTCGAAAACCCGAAACTGTGGAGCGCCGAAATCCCGAATCTCTATCGTGCGGTGGTTGAACTGCACACCGCCGACGGCACGC
GCTAGCGCAGTGTGATGCAGACTTGCAGCTTTTGGGCTTTGACACCTCGCGGCTTTAGGGCTTAGAGATAGCACGCCACCAACTTGACGTGTGGCGGCTGCCGTGCG

LacZ

1,180 1,200 1,220 1,240 1,260 1,280

HpaI

TGATTGAAGCAGAAGCCTGCGATGTCGGTTTCCGCGAGGTGCGGATTGAAAATGGTCTGCTGCTGCTGAACGGCAAGCCGTTGCTGATTCGAGGCGTTAACCGTCAC
ACTAACTTCGTCTTCGGACGCTACAGCCAAAGGCGCTCCACGCCTAACTTTTACCAGACGACGACGACTTGCCGTTCGGCAACGACTAAGCTCCGCAATTGGCAGTG

LacZ

1,300 1,320 1,340 1,360 1,380

EcoRV

GAGCATCATCCTCTGCATGGTCAGGTCATGGATGAGCAGACGATGGTGCAGGATATCCTGCTGATGAAGCAGAACAACTTTAACGCCGTGCGCTGTTCGCATTATCC
CTCGTAGTAGGAGACGTACCAGTCCAGTACCTACTCGTCTGCTACCACGTCCTATAGGACGACTACTTCGTCTTGTTGAAATTGCGGCACGCGACAAGCGTAATAGG

LacZ

1,400 1,420 1,440 1,460 1,480

DraIII SspI PflMI

GAACCATCCGCTGTGGTACACGCTGTGCGACCGCTACGGCCTGTATGTGGTGGATGAAGCCAATATTGAAACCCACGGCATGGTGCCAATGAATCGTCTGACCGATG
CTTGGTAGGCGACACCATGTGCGACACGCTGGCGATGCCGGACATACACCACCTACTTCGGTTATAACTTTGGGTGCCGTACCACGGTTACTTAGCAGACTGGCTAC

LacZ

1,500 1,520 1,540 1,560 1,580 1,600

BsoBI
AvaI BclI

ATCCGCGCTGGCTACCGGCGATGAGCGAACGCGTAACGCGAATGGTGCAGCGCGATCGTAATCACCCGAGTGTGATCATCTGGTCGCTGGGGAATGAATCAGGCCAC
TAGGCGCGACCGATGGCCGCTACTCGCTTGCGCATTGCGCTTACCACGTCGCGCTAGCATTAGTGGGCTCACACTAGTAGACCAGCGACCCCTTACTTAGTCCGGTG

LacZ

1,620 1,640 1,660 1,680 1,700

GGCGCTAATCACGACGCGCTGTATCGCTGGATCAAATCTGTCGATCCTTCCCGCCCGGTGCAGTATGAAGGCGGCGGAGCCGACACCACGGCCACCGATATTATTTG
CCGCGATTAGTGCTGCGCGACATAGCGACCTAGTTTAGACAGCTAGGAAGGGCGGGCCACGTCATACTTCCGCCGCCTCGGCTGTGGTGCCGGTGGCTATAATAAAC

LacZ

1,720 1,740 1,760 1,780 1,800

BssHII
MauBI BbsI

CCCGATGTACGCGCGCGTGGATGAAGACCAGCCCTTCCCGGCTGTGCCGAAATGGTCCATCAAAAAATGGCTTTCGCTACCTGGAGAGACGCGCCCGCTGATCCTTT
GGGCTACATGCGCGCGCACCTACTTCTGGTCGGGAAGGGCCGACACGGCTTTACCAGGTAGTTTTTTACCGAAAGCGATGGACCTCTCTGCGCGGGCGACTAGGAAA

LacZ

1,820 1,840 1,860 1,880 1,900 1,920

PflMI

GCGAATACGCCCACGCGATGGGTAACAGTCTTGGCGGTTTCGCTAAATACTGGCAGGCGTTTCGTCAGTATCCCCGTTTACAGGGCGGCTTCGTCTGGGACTGGGTG
CGCTTATGCGGGTGCGCTACCCATTGTCAGAACCGCCAAAGCGATTTATGACCGTCCGCAAAGCAGTCATAGGGGCAAATGTCCCGCCGAAGCAGACCCTGACCCAC

LacZ

1,940 1,960 1,980 2,000 2,020

GATCAGTCGCTGATTAAATATGATGAAAACGGCAACCCGTGGTCGGCTTACGGCGGTGATTTTGGCGATACGCCGAACGATCGCCAGTTCTGTATGAACGGTCTGGT
CTAGTCAGCGACTAATTTATACTACTTTTGCCGTTGGGCACCAGCCGAATGCCGCCACTAAAACCGCTATGCGGCTTGCTAGCGGTCAAGACATACTTGCCAGACCA

LacZ

2,040 2,060 2,080 2,100 2,120 2,140

AfeI

CTTTGCCGACCGCACGCCGCATCCAGCGCTGACGGAAGCAAAACACCAGCAGCAGTTTTTCCAGTTCCGTTTATCCGGGCAAACCATCGAAGTGACCAGCGAATACC

GAAACGGCTGGCGTGCGGCGTAGGTCGCGACTGCCTTCGTTTTGTGGTCGTCGTCAAAAAGGTCAAGGCAAATAGGCCCGTTTGGTAGCTTCACTGGTCGCTTATGG

LacZ

2,160 2,180 2,200 2,220 2,240

SacI
Eco53kI

TGTTCCGTCATAGCGATAACGAGCTCCTGCACTGGATGGTGGCGCTGGATGGTAAGCCGCTGGCAAGCGGTGAAGTGCCTCTGGATGTCGCTCCACAAGGTAAACAG

ACAAGGCAGTATCGCTATTGCTCGAGGACGTGACCTACCACCGCGACCTACCATTCGGCGACCGTTCGCCACTTCACGGAGACCTACAGCGAGGTGTTCCATTTGTC

LacZ

2,260 2,280 2,300 2,320 2,340

TTGATTGAACTGCCTGAACTACCGCAGCCGGAGAGCGCCGGGCAACTCTGGCTCACAGTACGCGTAGTGCAACCGAACGCGACCGCATGGTCAGAAGCCGGGCACAT

AACTAACTTGACGGACTTGATGGCGTCGGCCTCTCGCGGCCCGTTGAGACCGAGTGTCATGCGCATCACGTTGGCTTGCGCTGGCGTACCAGTCTTCGGCCCGTGTA

LacZ

2,360 2,380 2,400 2,420 2,440 2,460

BstXI
XcmI

CAGCGCCTGGCAGCAGTGGCGTCTGGCGGAAAACCTCAGTGTGACGCTCCCCGCCGCGTCCCACGCCATCCCGCATCTGACCACCAGCGAAATGGATTTTTGCATCG

GTCGCGGACCGTCGTCACCGCAGACCGCCTTTTGGAGTCACACTGCGAGGGGCGGCGCAGGGTGCGGTAGGGCGTAGACTGGTGGTCGCTTTACCTAAAAACGTAGC

LacZ

2,480 2,500 2,520 2,540 2,560

AGCTGGGTAATAAGCGTTGGCAATTTAACCGCCAGTCAGGCTTTCTTTCACAGATGTGGATTGGCGATAAAAAACAACTGCTGACGCCGCTGCGCGATCAGTTCACC

TCGACCCATTATTCGCAACCGTTAAATTGGCGGTCAGTCCGAAAGAAAGTGTCTACACCTAACCGCTATTTTTTGTTGACGACTGCGGCGACGCGCTAGTCAAGTGG

LacZ

2,580 2,600 2,620 2,640 2,660

CGTGCACCGCTGGATAACGACATTGGCGTAAGTGAAGCGACCCGCATTGACCCTAACGCCTGGGTCGAACGCTGGAAGGCGGCGGGCCATTACCAGGCCGAAGCAGC

GCACGTGGCGACCTATTGCTGTAACCGCATTCACTTCGCTGGGCGTAACTGGGATTGCGGACCCAGCTTGCGACCTTCCGCCGCCCGGTAATGGTCCGGCTTCGTCG

LacZ

2,680 2,700 2,720 2,740 2,760 2,780

GTTGTTGCAGTGCACGGCAGATACACTTGCTGATGCGGTGCTGATTACGACCGCTCACGCGTGGCAGCATCAGGGGAAAACCTTATTTATCAGCCGGAAAACCTACC

CAACAACGTCACGTGCCGTCTATGTGAACGACTACGCCACGACTAATGCTGGCGAGTGCGCACCGTCGTAGTCCCCTTTTGGAATAAATAGTCGGCCTTTTGGATGG

LacZ

2,800 2,820 2,840 2,860 2,880

BspMI
BfuAI

```
GGATTGATGGTAGTGGTCAAATGGCGATTACCGTTGATGTTGAAGTGGCGAGCGATACACCGCATCCGGCGCGGATTGGCCTGAACTGCCAGCTGGCGCAGGTAGCA
CCTAACTACCATCACCAGTTTACCGCTAATGGCAACTACAACTTCACCGCTCGCTATGTGGCGTAGGCCGCGCCTAACCGGACTTGACGGTCGACCGCGTCCATCGT
```

LacZ

2,900 2,920 2,940 2,960 2,980

AccI
BstZ17I
PciI BbsI

```
GAGCGGGTAAACTGGCTCGGATTAGGGCCGCAAGAAAACTATCCCGACCGCCTTACTGCCGCCTGTTTTGACCGCTGGGATCTGCCATTGTCAGACATGTATACCCC
CTCGCCCATTTGACCGAGCCTAATCCCGGCGTTCTTTTGATAGGGCTGGCGGAATGACGGCGGACAAAACTGGCGACCCTAGACGGTAACAGTCTGTACATATGGGG
```

LacZ

3,000 3,020 3,040 3,060 3,080 3,100

BsoBI
BsiWI AvaI BstXI

```
GTACGTCTTCCCGAGCGAAAACGGTCTGCGCTGCGGGACGCGCGAATTGAATTATGGCCCACACCAGTGGCGCGGCGACTTCCAGTTCAACATCAGCCGCTACAGTC
CATGCAGAAGGGCTCGCTTTTGCCAGACGCGACGCCCTGCGCGCTTAACTTAATACCGGGTGTGGTCACCGCGCCGCTGAAGGTCAAGTTGTAGTCGGCGATGTCAG
```

LacZ

3,120 3,140 3,160 3,180 3,200

XcmI
NdeI PfoI

```
AACAGCAACTGATGGAAACCAGCCATCGCCATCTGCTGCACGCGGAAGAAGGCACATGGCTGAATATCGACGGTTTCCATATGGGGATTGGTGGCGACGACTCCTGG
TTGTCGTTGACTACCTTTGGTCGGTAGCGGTAGACGACGTGCGCCTTCTTCCGTGTACCGACTTATAGCTGCCAAAGGTATACCCCTAACCACCGCTGCTGAGGACC
```

LacZ

3,220 3,240 3,260 3,280 3,300

EcoRI
ApoI BlpI

```
AGCCCGTCAGTATCGGCGGAATTCCAGCTGAGCGCCGGTCGCTACCATTACCAGTTGGTCTGGTGTCAAAAATAAttaagccagccccgacacccgccaacacccgc
TCGGGCAGTCATAGCCGCCTTAAGGTCGACTCGCGGCCAGCGATGGTAATGGTCAACCAGACCACAGTTTTTATTaattcggtcggggctgtgggcggttgtgggcg
```

LacZ

3,320 3,340 3,360 3,380 3,400 3,420

PfoI

```
tgacgcgccctgacgggcttgtctgctcccggcatccgcttacagacaagctgtgaccgtctccgggagctgcatgtgtcagaggttttcaccgtcatcaccgaaac
actgcgcgggactgcccgaacagacgagggccgtaggcgaatgtctgttcgacactggcagaggccctcgacgtacacagtctccaaaagtggcagtagtggctttg
```

3,440 3,460 3,480 3,500 3,520

```
                                                                                AatII
                   EcoO109I                                                   ZraI
gcgcgagacgaaagggcctcgtgatacgcctatttttataggttaatgtcatgataataatggtttcttagacgtcaggtggcacttttcggggaaatgtgcgcgga
cgcgctctgctttcccggagcactatgcggataaaaatatccaattacagtactattattaccaaagaatctgcagtccaccgtgaaaagcccctttacacgcgcct
        3,540               3,560               3,580               3,600               3,620

                                                                                         SspI
acccctatttgtttatttttctaaatacattcaaatatgtatccgctcatgagacaataaccctgataaatgcttcaataatattgaaaaaggaagagtatgagtat
tggggataaacaaataaaaagatttatgtaagtttatacataggcgagtactctgttattgggactatttacgaagttattataactttttccttctcatactcata
                                         AmpR promoter
  3,640               3,660               3,680               3,700               3,720               3,740

tcaacatttccgtgtcgcccttattcccttttttgcggcattttgccttcctgtttttgctcacccagaaacgctggtgaaagtaaaagatgctgaagatcagttgg
agttgtaaaggcacagcgggaataagggaaaaaacgccgtaaaacggaaggacaaaaacgagtgggtctttgcgaccactttcattttctacgacttctagtcaacc
                                                       AmpR
              3,760               3,780               3,800               3,820               3,840

                                                                              XmnI
                                                                            AclI
gtgcacgagtgggttacatcgaactggatctcaacagcggtaagatccttgagagttttcgccccgaagaacgttttccaatgatgagcacttttaaagttctgcta
cacgtgctcacccaatgtagcttgacctagagttgtcgccattctaggaactctcaaaagcggggcttcttgcaaaaggttactactcgtgaaaatttcaagacgat
                                                                                                 AmpR
                                                       AmpR
       3,860               3,880               3,900               3,920               3,940

                                                                                            ScaI
                                                                                          TatI
tgtggcgcggtattatcccgtattgacgccgggcaagagcaactcggtcgccgcatacactattctcagaatgacttggttgagtactcaccagtcacagaaaagca
acaccgcgccataatagggcataactgcggcccgttctcgttgagccagcggcgtatgtgataagagtcttactgaaccaactcatgagtggtcagtgtcttttcgt
                                                       AmpR
                                                       AmpR
  3,960               3,980               4,000               4,020               4,040               4,060

tcttacggatggcatgacagtaagagaattatgcagtgctgccataaccatgagtgataacactgcggccaacttacttctgacaacgatcggaggaccgaaggagc
agaatgcctaccgtactgtcattctcttaatacgtcacgacggtattggtactcactattgtgacgccggttgaatgaagactgttgctagcctcctggcttcctcg
                                                       AmpR
                                                       AmpR
            4,080               4,100               4,120               4,140               4,160
```

```
taaccgctttttttgcacaacatgggggatcatgtaactcgccttgatcgttgggaaccggagctgaatgaagccataccaaacgacgagcgtgacaccacgatgcct
attggcgaaaaaacgtgttgtaccccctagtacattgagcggaactagcaacccttggcctcgacttacttcggtatggtttgctgctcgcactgtggtgctacgga
AmpR
AmpR
4,180   4,200   4,220   4,240   4,260   4,280

        AclI
BsrDI        FspI                                                        NmeAIII
gtagcaatggcaacaacgttgcgcaaactattaactggcgaactacttactctagcttcccggcaacaattaatagactggatggaggcggataaagttgcaggacc
catcgttaccgttgttgcaacgcgtttgataattgaccgcttgatgaatgagatcgaagggccgttgttaattatctgacctacctccgcctatttcaacgtcctgg
AmpR
AmpR
4,300   4,320   4,340   4,360   4,380

                                                                BsaI BsrDI
acttctgcgctcggcccttccggctggctggtttattgctgataaatctggagccggtgagcgtgggtctcgcggtatcattgcagcactggggccagatggtaagc
tgaagacgcgagccgggaaggccgaccgaccaaataacgactatttagacctcggccactcgcacccagagcgccatagtaacgtcgtgaccccggtctaccattcg
AmpR
AmpR
4,400   4,420   4,440   4,460   4,480

                              AhdI
cctcccgtatcgtagttatctacacgacggggagtcaggcaactatggatgaacgaaatagacagatcgctgagataggtgcctcactgattaagcattggtaactg
ggagggcatagcatcaatagatgtgctgcccctcagtccgttgatacctacttgctttatctgtctagcgactctatccacggagtgactaattcgtaaccattgac
AmpR
AmpR
4,500   4,520   4,540   4,560   4,580   4,600

tcagaccaagtttactcatatatactttagattgatttaaaacttcatttttaatttaaaaggatctaggtgaagatcctttttgataatctcatgaccaaaatccc
agtctggttcaaatgagtatatatgaaatctaactaaattttgaagtaaaaattaaattttcctagatccacttctaggaaaaactattagagtactggttttaggg
ColE1...igin
4,620   4,640   4,660   4,680   4,700

ttaacgtgagttttcgttccactgagcgtcagaccccgtagaaaagatcaaaggatcttcttgagatcctttttttctgcgcgtaatctgctgcttgcaaacaaaaa
aattgcactcaaaagcaaggtgactcgcagtctggggcatcttttctagtttcctagaagaactctaggaaaaaaagacgcgcattagacgacgaacgtttgttttt
ColE1 origin
4,720   4,740   4,760   4,780   4,800

aaccaccgctaccagcggtggtttgtttgccggatcaagagctaccaactctttttccgaaggtaactggcttcagcagagcgcagataccaaatactgttcttcta
ttggtggcgatggtcgccaccaaacaaacggcctagttctcgatggttgagaaaaaggcttccattgaccgaagtcgtctcgcgtctatggtttatgacaagaagat
ColE1 origin
4,820   4,840   4,860   4,880   4,900   4,920
```

gtgtagccgtagttaggccaccacttcaagaactctgtagcaccgcctacatacctcgctctgctaatcctgttaccagtggctgctgccagtggcgataagtcgtg
cacatcggcatcaatccggtggtgaagttcttgagacatcgtggcggatgtatggagcgagacgattaggacaatggtcaccgacgacggtcaccgctattcagcac
ColE1 origin
4,940 4,960 4,980 5,000 5,020

tcttaccgggttggactcaagacgatagttaccggataaggcgcagcggtcgggctgaacggggggttcgtgcacacagcccagcttggagcgaacgacctacaccg
agaatggcccaacctgagttctgctatcaatggcctattccgcgtcgccagcccgacttgccccccaagcacgtgtgtcgggtcgaacctcgcttgctggatgtggc
ColE1 origin
5,040 5,060 5,080 5,100 5,120

aactgagatacctacagcgtgagctatgagaaagcgccacgcttcccgaagggagaaaggcggacaggtatccggtaagcggcagggtcggaacaggagagcgcacg
ttgactctatggatgtcgcactcgatactctttcgcggtgcgaagggcttccctctttccgcctgtccataggccattcgccgtcccagccttgtcctctcgcgtgc
ColE1 origin
5,140 5,160 5,180 5,200 5,220 5,240

agggagcttccagggggaaacgcctggtatctttatagtcctgtcgggtttcgccacctctgacttgagcgtcgatttttgtgatgctcgtcaggggggcggagcct
tccctcgaaggtccccctttgcggaccatagaaatatcaggacagcccaaagcggtggagactgaactcgcagctaaaaacactacgagcagtccccccgcctcgga
ColE1 origin
5,260 5,280 5,300 5,320 5,340

PciI
atggaaaaacgccagcaacgcggcctttttacggttcctggccttttgctggccttttgctcacatgttctttcctgcgttatcccctgattctgtggataaccgta
taccttttttgcggtcgttgcgccggaaaaatgccaaggaccggaaaacgaccggaaaacgagtgtacaagaaaggacgcaataggggactaagacacctattggcat
ColE1 origin
5,360 5,380 5,400 5,420 5,440

BspQI
SapI
ttaccgcctttgagtgagctgataccgctcgccgcagccgaacgaccgagcgcagcgagtcagtgagcgaggaagcggaaga
aatggcggaaactcactcgactatggcgagcggcgtcggcttgctggctcgcgtcgctcagtcactcgctccttcgccttct
5,460 5,470 5,480 5,490 5,500 5,510 5,520 5,530
